# Convergent evolution of face spaces across human face-selective neuronal groups and deep convolutional networks

**DOI:** 10.1038/s41467-019-12623-6

**Published:** 2019-10-30

**Authors:** Shany Grossman, Guy Gaziv, Erin M. Yeagle, Michal Harel, Pierre Mégevand, David M. Groppe, Simon Khuvis, Jose L. Herrero, Michal Irani, Ashesh D. Mehta, Rafael Malach

**Affiliations:** 1 Department of Neurobiology, Weizmann Institute of Science, 76100 Rehovot, Israel; 2 Department of Computer Science and Applied Mathematics, Weizmann Institute of Science, 76100 Rehovot, Israel; 3 Department of Neurosurgery, Donald and Barbara Zucker School of Medicine at Hofstra/Northwell and Feinstein Institute for Medical Research, Manhasset, NY 11030 USA; 4 Neurology Division, Clinical Neuroscience Department, Geneva University Hospital and Faculty of Medicine, Geneva, 1205 Switzerland; 5 The Krembil Neuroscience Centre, Toronto, ON M5T 2S8 Canada

**Keywords:** Perception, Extrastriate cortex

## Abstract

The discovery that deep convolutional neural networks (DCNNs) achieve human performance in realistic tasks offers fresh opportunities for linking neuronal tuning properties to such tasks. Here we show that the face-space geometry, revealed through pair-wise activation similarities of face-selective neuronal groups recorded intracranially in 33 patients, significantly matches that of a DCNN having human-level face recognition capabilities. This convergent evolution of pattern similarities across biological and artificial networks highlights the significance of face-space geometry in face perception. Furthermore, the nature of the neuronal to DCNN match suggests a role of human face areas in pictorial aspects of face perception. First, the match was confined to intermediate DCNN layers. Second, presenting identity-preserving image manipulations to the DCNN abolished its correlation to neuronal responses. Finally, DCNN units matching human neuronal group tuning displayed view-point selective receptive fields. Our results demonstrate the importance of face-space geometry in the pictorial aspects of human face perception.

## Abstract (web summary)

Deep convolutional neural networks (DCNNs) are able to identify faces on par with humans. Here, the authors record neuronal activity from higher visual areas in humans and show that face-selective responses in the brain show similarity to those in the intermediate layers of the DCNN.

## Introduction

Systems neuroscience research has been one of the fastest growing fields of science in recent years, accumulating detailed depictions of neuronal functional properties. However, despite this progress, two fundamental questions remain unsolved. First, we remain largely in the dark regarding how the different functional selectivities of individual neurons integrate in producing the observed cognitive and behavioral tasks. Second, with the exception of relatively peripheral neuronal circuits (e.g. directional selectivity in the retina^1^), no realistic wiring diagram is available to demonstrate how higher order neuronal tuning curves may actually be generated through neuronal network connectivity.

Considering the example of the ventral stream visual recognition system, an invaluable body of research has been obtained, depicting in great detail the neuronal selectivities in face-selective areas in monkeys^2^ and in humans^3,4^. Furthermore, clinical^5^ as well as causal manipulation evidence demonstrated the significance of these regions to face perception and identification^6–10^. However, the precise manner by which the neuronal tuning profiles of face-selective populations combine to allow face discrimination and person recognition are not yet fully understood, with various hypotheses and classification models accounting for a limited set of observations^11–13^. It is also not clear that classifiers capable of decoding faces from neuronal activity patterns truly reflect how the brain actually employs this neuronal activity for face perception^14^. These limitations are not unique to visual processes. In fact, all attempts to model high-level neuronal properties rely on limited models that can only be loosely linked to perception and behavior. The problem is largely due to the lack of models whose functional performance can achieve realistic human or animal levels^15^.

This problematic situation has been transformed in the last few years with the discovery that artificial Deep Convolutional Neural Networks (DCNNs) can now approach human-level performance in a variety of visual tasks, for example in face recognition^16–18^. This rapidly unfolding revolution offers the field of systems neuroscience a new type of models that achieve realistic human performance in specific tasks. Indeed, a number of recent studies have provided encouraging indications for the usefulness of DCNNs in predicting visual responses along the human visual hierarchy^19–21^, as well as in capturing category-selective representational geometries in visual cortex of humans and monkeys^22–24^.

A particularly interesting line of such modeling is the attempt to find aspects of convergent evolution, i.e. similar functional properties between artificial and biological systems^20^. The rational here is that if two very different systems that solve a similar functional task—face recognition in our case—develop certain similar characteristics, this may point to the importance of these characteristics in accomplishing the task. This can be nicely illustrated in the convergent evolution of wings across insects, birds, and even mammals—and most strikingly in their appearance in man-made airplanes. The important point to note is that in deducing function from convergent evolution, the farther the two converging systems are from each other, the more compelling is the role of the independently evolved property in these systems.

Here, we employed this approach to explore the hypothesis, originally proposed by Edelman and Grill-Spector^25^, that the unique structure of the face-space geometry, as defined by pair-wise similarities in activation patterns to different face images, constitutes a critical aspect in face perception. Such pattern similarity analysis, also termed Representation Similarity Analysis (RSA)^26^, has been successfully employed in comparing activations across visual categories between species^27^, and also in relation to DCNNs^22^.

Our results reveal a significant and consistent similarity between the face-space geometries of human cortical face selective neuronal groups and that of an artificial DCNN achieving human-level face recognition performance (VGG-Face^17^). We further used the DCNN correlation to examine whether the tuning properties of face-selective neuronal groups reflect a pictorial or person identity representation. In an identity representation, a neuron is predicted to be invariant to viewpoint or appearance changes of a person’s face, as long as the identity is preserved. By contrast, a neuron that is part of a pictorial representation of a face will modulate its activity following such identity-preserving changes. Our results suggest a high-level pictorial function for neuronal face representations since targeted image manipulations revealed invariance of the matching DCNN layers when presented with changes in low-level features (background removal, gray scale conversion, and luminance changes) but shifting image viewpoints, without affecting the identity, significantly reduced pair-wise similarities in the DCNN layers, as well as the DCNN to neural match. Finally, receptive fields of individual DCNN units that were found to match specific face neuronal groups displayed view-specific face fragments such as ears and eyes^28^.

Together, these findings highlight the power of DCNNs as a productive model of neuronal function. They further argue for the importance of face-space geometry in enabling face perception and they support, at least for the 1-back task employed in the study, a pictorial function of high-order face-selective regions of the human visual cortex.

## Results

### Face-selective contacts detected in three experimental tasks

**Fig. 1:**
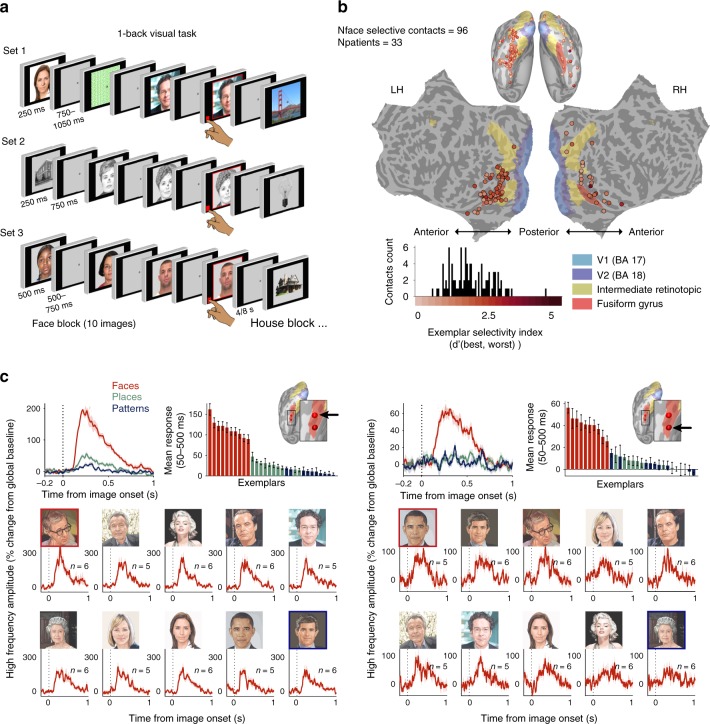
Experimental design, localization and exemplar selectivity of face selective iEEG contacts. **a** Schematic illustration of the 1-back visual tasks. Three versions, each including a different set of face stimuli and additional images from other categories, were included in the study. Every patient took part in either one or two versions out of the three. Faces used in set 3 (examples shown here) were taken from the face database by Minear and Park^56^. **b** Anatomical distribution of detected face contacts, projected onto a common template of the inflated (ventral view, top) and flattened cortex. Color coding denotes exemplar selectivity indices, defined as the *d*′ between the most- and the least-preferred face exemplars. Histogram presents the distribution of selectivity indices alongside the color code bar. The same distribution is presented separately for each of the three sets in Supplementary Fig. 1. **c** Example of two neighboring face contacts implanted in the same patient, their anatomical locations marked by black arrows on the inflated left hemisphere. Top left panel depicts the mean HFA response across face, place, and pattern exemplars in set 1, demonstrating robust face selectivity. Top right bar plot depicts the mean HFA response (50–500 ms) to each of the exemplars in a descending order of activation, with bar colors denoting visual category. Bottom galleries present the mean HFA response of each contact to the different faces with the corresponding images. Galleries are presented in a descending order of mean response (as in the bar plots). HFA was normalized to % signal change from global baseline at −200 to 0 ms. All error bars denote s.e.m. Note the differences in response amplitude to different exemplars which was unique to each iEEG contact. Faces shown for sets 1 and 2 in all figures are accurate illustrations of the original images used, in compliance with copy rights limitations. Exceptions are Fig. 4 and Supplementary Fig. 12, in which original images of most exemplars in set 1 are shown

Visual responses to three independent sets of images, including human faces and four or five additional categories, were recorded using either subdural or depth intracranial EEG (iEEG) electrodes (see Methods for stimuli and recording details). Figure 1a depicts the three versions of a 1-back experimental design in which patients were instructed to view images, presented 3–6 times in pseudo-random order, for 250 ms (sets 1–2) or 500 ms (set 3) and press a button for image repeats. We focused on the high frequency amplitude (HFA, 48–154 Hz) signal, previously shown to reveal functional-selectivity^29,30^, and to constitute a reliable index of aggregate firing rate in humans^31,32^. Altogether, 8916 contacts in 61 patients were analyzed. Face contacts were defined as having significantly higher HFA response to faces relative to places and to patterns (two paired *t*-tests, pFDR < 0.05). In total, 96 contacts (33 patients) were found to be face selective. Figure 1b depicts the cortical distribution of these face selective contacts which, as can be seen, were concentrated mainly in the high-order ventral visual cortex (see Supplementary Fig. 1 for the separate sets).

### Exemplar selectivity of face selective contacts

Examination of the responses to individual face exemplars in each face contact revealed substantial differences in activation amplitudes across exemplars. This can be readily discerned in the gallery of responses obtained from a single contact shown in the left bottom of Fig. 1c, where, for example, the response to the image of Woody Allen was more than 1.5 times greater than the response to Obama. To quantify this phenomenon, we defined an exemplar selectivity index for each face contact (*d*′ between preferred and least-preferred exemplars). The distribution of exemplar selectivity indices and their corresponding anatomical sites are depicted in Fig. 1b and in Supplementary Fig. 1. All face contacts showed a significant level of exemplar selectivity (image permutation test per contact, all pFDR < 0.01). Importantly, the specific profile of exemplar selectivity changed across neighboring contacts (see Fig. 1c for an example of the different profiles in two neighboring contacts).

At the level of contacts’ ensemble, such heterogeneous exemplar selectivity in single face contacts could potentially underlie neural discriminability between individual faces. To examine this possibility, we applied a simple pattern-matching decoding analysis (see Methods and Supplementary Fig. 2a for a schematic illustration of decoding procedure). As presented in Supplementary Fig. 2b., decoding accuracy was significantly above chance in sets 1 and 2 (image permutation test: set 1: *p* = 0.001; set 2: *p* = 0.01). Set 3 showed only a positive trend, likely due to a smaller number of patients who took part in the third task version and consequently the smaller number of face contacts included in this set (*p* = 0.24).

### Match between neural and DCNN face-spaces

**Fig. 2:**
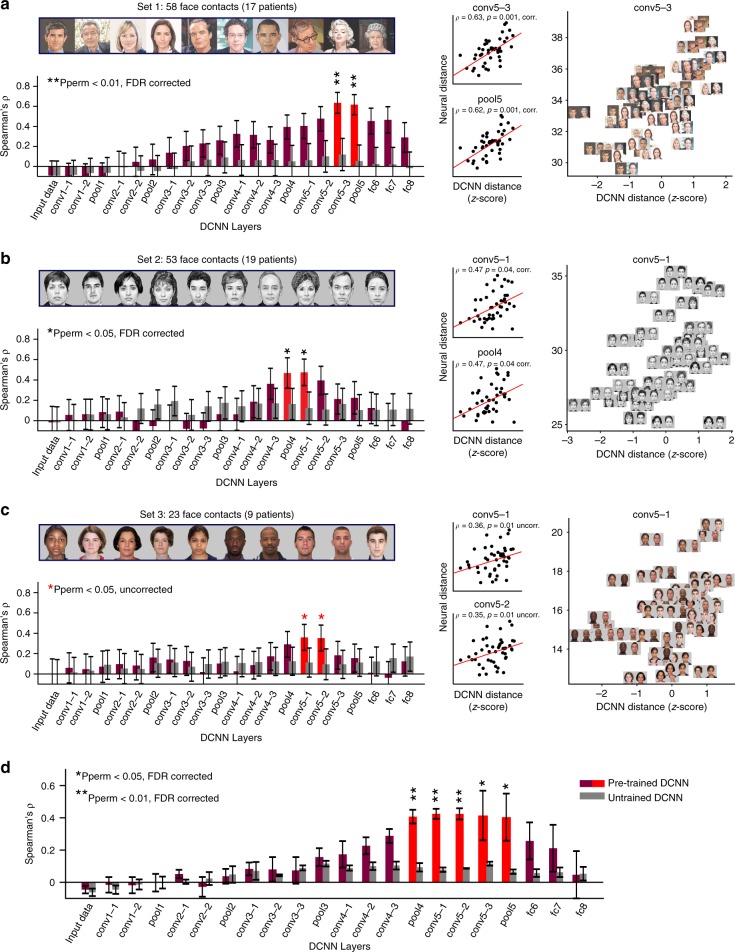
Pair-wise distances in the neural face-space match the same distances in intermediate DCNN layers. **a** Left: Purple and red bars denote the correlation between pair-wise distances in iEEG face-selective contacts included in set 1 and in the different layers of a DCNN pre-trained on face recognition (VGG-Face). Gray bars denote the same correlations, but to untrained VGG-Face layers. Face exemplars included in set 1 are presented above the bar plot. Error bars denote image pairs bootstrap s.e.m. Middle: Scatter plots which underlie the correlation in the significantly correlated DCNN layers. Red line is the least-squares linear regression fit. Right: Enlarged scatter plot for the maximally correlated DCNN layer (same data as in the top scatter plot in middle panel), with images of face pairs presented on top of individual dots. **b**, **c** Same as panel **a**, only for sets 2 and 3, respectively. Faces used in set 3 (and shown here in panel **c**) were taken from the face database by Minear and Park^56^ . Note that each set consisted of a different (yet partially overlapping) group of face contacts given the group of patients who participated in the corresponding task version. **d** Weighted averages of the correlation coefficients observed across the three sets. Sets were weighted by the number of face contacts they included. Error bars denote the weighted s.e.m. across the sets. Note the consistent correlation of the iEEG recordings to mid-level DCNN layers. All *p* values were derived from an image permutation test (1000 permutations). Reported *p* values are FDR corrected, except for set 3 in which significance did not survive FDR correction

What could be the function of the observed face-exemplar selectivity? To find out whether a DCNN with human-level face recognition performance (VGG-Face) could serve as a realistic functional model of these selectivities, we examined whether the face-space geometry of face exemplars, as determined by pair-wise distances between their activation patterns^25,26^, was similar between the human cortex and individual DCNN layers. Pair-wise activation distances were measured for all face exemplar pairs both in the human cortex and in each of the VGG-Face layers. For the neural data, we defined an activity pattern per exemplar as the vector of concatenated responses at 50–500 ms obtained from all face contacts in the relevant set (response time series was averaged across repetitions). A pair-wise distance between two exemplars was defined as the Euclidean distance between the two response vectors generated by a pair of faces. Comparing all pair-wise distances generated by the iEEG recordings with those generated by VGG-Face when presented with the same face images revealed a significant correlation that was consistent across the three sets. Importantly, significant correlations were limited only to the intermediate DCNN layers, and chance performance was evident at early, low-level feature-selective layers of the hierarchy and at the top, identity-selective layers. Figure 2 shows the correlation between each iEEG data set and each DCNN layer (Fig. 2 left), the scatter plots of the pair-wise distances for the two maximally correlated layers in each set (Fig. 2 middle) and the actual pairs of face exemplars presented on top of the scatter plot from the maximally correlated layer in each set (Fig. 2 right). The correlation between iEEG recordings and two specific intermediate layers was significant in the first two sets (image permutation test followed by FDR correction across layers; all pFDR < 0.05; see Fig. 2 for correlation coefficients and *p* values) and a similar trend was observed for two intermediate layers in the third set, which included only 23 face contacts and also showed the weakest exemplar decoding performance (image permutation test, *p* < 0.05, uncorrected).

We further performed a pooled analysis to assess the consistency of the match across sets. Figure 2d depicts the weighted-mean of correlations across the three sets for every DCNN layer, with set weights assigned according to the number of face contacts. Five intermediate layers, ranging from layer pool4 to pool5, significantly matched the neural distances (image permutation test followed by FDR correction, all pFDR < 0.05).

To directly examine the nature of representation in these five intermediate layers, we examined their sensitivity to changing the view point of faces while preserving their person identity. We generated the pair-wise distance matrix between frontal and full profile views (conducted on 65 independent identities from the KDEF database^33^) for each of the five DCNN layers, as well as for the subsequent three fully connected layers (fc6–fc8). The diagonal in such a matrix represents the representational distance between frontal and profile view of the same identities, with low values indicating viewpoint invariance, i.e. that the layer is able to generalize person identity across view point rotations. As can be seen in Supplementary Fig. 3, such low values along the diagonal (dark pixels) were rare in the five intermediate layers matching the neural face-space, and emerged only in the subsequent three fully connected layers.

Was the effect a result of pre-training the artificial network on a large set of face exemplars? To examine this, we ran the same analysis on an untrained VGG-Face network, preserving the same architecture while assigning its connections random weights (xavier normal initialization). Randomizing the connection weights resulted in a near complete abolishment of the correlation to the neural face-space (Fig. 2, gray bars).

The high temporal resolution of the iEEG signal enabled us to investigate whether the maximally correlated DCNN layers were altered throughout the neural response. To this end, we computed the maximally correlated DCNN layer at a sliding window of 200 ms, with a 50 ms stride. The results (Supplementary Fig. 4) revealed a consistent assignment of the same layers observed in the original analysis which focused on a single time window at 50–500 ms. Thus, we have found no evidence for an evolution in the highest matching DCNN layer across time, at least for up to 500 ms presentation durations.

We further examined whether the effect was present also at the individual patient level. To this end, we recomputed the correlations separately in individual patients that had a minimum of five face contacts, and averaged the individual correlational patterns (13 patterns in total). The resultant average correlational pattern, presented in Supplementary Fig. 5, followed a similar pattern to that found when pooling face contacts from all patients but with somewhat lower effect sizes, likely due to the reduction in the number of contacts.

To ensure the correlation was not confounded by differences in low-level image parameters between face exemplars, we also computed the partial correlation between neural and DCNN face spaces (reflected in pair-wise distances) in each of the three sets, while partialing out the potential contribution of pair-wise distances between luminance, saturation, mean gradient, and RMS contrast on the observed match. We found no significant change in any of the three sets (see Methods and Supplementary Fig. 6), arguing against low-level variations between images confounding the brain to DCNN match.

We next tested whether the temporal dynamics in the neural responses contribute to the observed neural to DCNN match. The same analysis presented in Fig. 2 was conducted while averaging the response across time (50–500 ms) to eliminate the information contained in the dynamic response profile. Despite averaging the dynamic profile, correlations in all three sets remained significant in the same layers as prior to averaging (Supplementary Fig. 7). This result suggests that the precise dynamical wave-form was not critical in generating the distance correlations to the DCNN layers.

Was the observed correlation dependent on training the network specifically on face recognition? Comparing the distance matrices in the same manner but this time using a different network—VGG-16^34^, trained to categorize objects rather than faces^35^, revealed that the average correlation across the three sets to VGG-16 followed a similar pattern to that of VGG-face (Supplementary Fig. 8). This result suggests that training on the specific task of face identification is not necessary to achieve a representational geometry that matches the neural face-space.

### Low- and high-level manipulations on DCNN input images

**Fig. 3:**
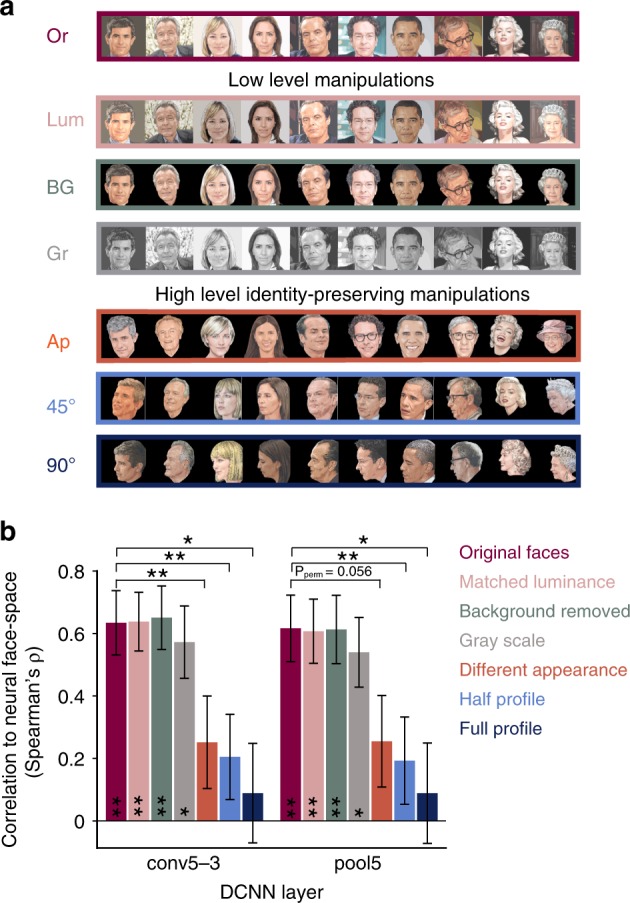
The impact of low- and high-level manipulations on the DCNN to neural face-space match. **a** Manipulated face exemplars included in set 1, from top to bottom: Or - original face images, Lum - matched luminance, BG - removed background, Gr - gray scale conversion, Ap - different appearance of the same identities which differ in pictorial aspects such as facial expression and haircut, 45° and 90° - different images of the same identities, rotated at ~45° and ~90° view-points, respectively. **b** The impact of feeding the manipulated images presented in panel **a** to the DCNN on its correlation to neural distances in layers that originally showed a significant correlation in set 1 (layers conv5–3 and pool5). Error bars denote bootstrap s.e.m. on image pairs. Whereas low-level manipulations did not impact the correlation to neural data, higher level yet identity-preserving manipulations significantly reduced the correlation to chance. *P* values were defined as the proportion of correlations delta for shuffled image labels (1000 permutations) that exceeded the delta obtained from correctly labeled data. *p < 0.05, **p < 0.01. A 95% image bootstrap confidence interval test around the original correlation value yielded the same significance profile across manipulations. The differential sensitivity to image manipulations points to a high-level pictorial rather than personal-identity function of the iEEG recorded face neuronal groups

The availability of a functional model of the neural face-space makes it possible to perform controlled manipulations on the images presented to the network and examine their impact on its internal representation and on its match to the neural data. Figure 3 compares the correlation to neural data in set 1 given the original images as DCNN input with the correlation after applying six types of manipulations to the images presented to the DCNN. Three manipulations addressed the issue of low-level features: matching the overall luminance of the images (pink bar), converting the background of the face images to black (turquoise bars), and converting the colored faces to gray scale (gray bar). These low-level manipulations had no significant effect on the brain to DCNN correlations observed in sets 1 to 3 (95% image bootstrap confidence interval test around the original correlation value, and a permutation test on original correlation delta against those obtained from 1000 image permutations, all *p* > 0.27; see Supplementary Fig. 9 for sets 2 and 3).

Conversely, presenting the DCNN with the same identities but in a different appearance (e.g. facial expression, haircut), and the same identities in similar appearance but with partial (~45°) head rotation—manipulations that change pictorial aspects while maintaining personal identity—significantly reduced the correlation (pale orange bar and blue bar, respectively). Moreover, a ~90° rotation to a profile view completely abolished the correlation (dark blue bar). Note that these identity-preserving manipulations of the face exemplars were available only for set 1, consisting of famous faces.

Comparing the pair-wise distances in the same DCNN layers when presented with the original and manipulated images as inputs yielded essentially similar results (Supplementary Fig. 11a): low-level manipulations had a marginal impact on the pair-wise distances in these DCNN layers (Spearman’s rho ranged from 0.97 to 0.93), whereas high-level manipulations significantly decreased the correlations (Spearman’s rho ranged from 0.59 to 0.37; image permutation test on correlations delta, all *p* < 0.05).

Could it be that the DCNN as a whole was unable to generalize over view-points or appearance changes in the specific set of images used in our study? Mean decoding accuracy of identities across the high-level manipulations was 92% for the top fully connected layer (fc8) but dropped to 63% and 66% for the layers that matched the neural data (conv5–3 and pool5, respectively; see Supplementary Fig. 10 and Methods), suggesting that identity generalization can be achieved in the top DCNN layer. Further, mean response amplitude of the relevant DCNN layers following high-level manipulations remained unchanged (two-tailed Wilcoxon signed-rank test; all *p* > 0.07; Supplementary Fig. 11b). Plotting the activity distribution of units in the two layers following all six image manipulations revealed that they remained largely unaffected (Supplementary Fig. 11c), while the identity of activated units was substantially altered only following the three high-level manipulations (Supplementary Fig. 11d).

### Receptive field visualization of model units

**Fig. 4:**
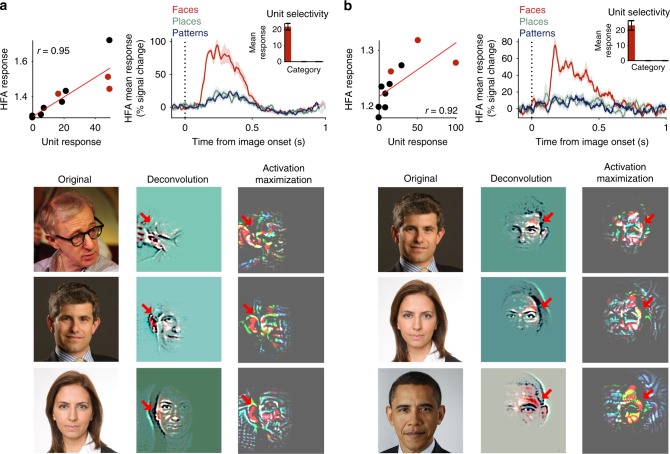
Receptive field reconstructions of two example model units. Two out of the seven model units that were found to significantly predict responses of single face contacts in a leave-one out search (see Supplementary Fig. 12 for the full set). Model units were defined as units which were best correlated with a single iEEG contact responses to all *N*−1 exemplar sets and significantly predicted the held out responses based on a linear fit. **a** Model unit found in layer pool5 for set 1. Top left scatter plot depicts the responses of the model unit versus the mean HFA responses (50–500 ms) of the face contact. Red dots correspond to the three exemplars for which receptive field visualizations are presented (see below). Red line is the least-squares linear fit. Top right plot depicts the mean categorical responses of the face-selective contact to which the model unit matched. Bar inset presents the categorical selectivity of the model unit, which responded solely to faces and not to places or patterns. All error bars  denote s.e.m. across exemplars. Bottom gallery presents visualizations of the model unit’s receptive field for three example face exemplars: left column shows the original images in a descending order of the model unit’s response; middle column shows the reconstructed receptive field using deconvolution; right column shows the delta between the altered image following activation maximization and the original image, reflecting the added image that maximally amplified the model unit’s response. For visualization purpose, the contrast of each receptive field image was normalized by stretching the active range of RGB values. **b** Same as panel **a**, depicting a different model unit detected in layer pool5 for set 1 and its matching face contact. Note the view point selectivity and consistency of the face fragments that are highlighted by the two visualization procedures, and the large expanse of image changes following activation maximization. Copy rights holders: Woodie Allen photo—Colin Swan, license link https://creativecommons.org/licenses/by-sa/2.0/; Christopher Falkenberg photo—Christopher Falkenberg; Gila Gamliel photo—https://en.wikipedia.org/wiki/Gila_Gamliel#/media/File:Gila_Gamliel.jpg, license link https://creativecommons.org/licenses/by-sa/3.0/deed.en; Barack Obama photo—Pete Souza, license link https://creativecommons.org/licenses/by/3.0/deed.en

The match of the neural face-space to specific DCNN layers opens the possibility of modeling the actual tuning properties of individual face contacts and exploring putative optimal receptive field properties that may account for their tuning properties. By contrast to the RSA method, this approach assumes a first order similarity between tuning curves of single contacts and individual artificial units. To examine such putative model units, we implemented a leave-one out cross-validation search in the relevant DCNN layers to detect single artificial units that could significantly predict the responses of specific face contacts to different face exemplars (see Methods). Our results uncovered a small set of such artificial units (cluster correction applied, see Methods). Every detected model unit was found to significantly predict responses of a face contact to the held out exemplars based on a linear fit. Figure 4 depicts two examples of such model units. The top left scatter plot in each panel depicts the correlation between responses to face exemplars in the model unit (*y*-axis) and in the face contact (*x*-axis), reflecting the significant similarity between the two.

We then took the advantage of DCNN modeling to actually map the optimal stimuli, i.e. receptive field, of the model units. We applied two different methods for such mapping: deconvolution (also termed transposed convolution) and activation maximization (see Methods for details). Applying deconvolution on a model unit given its response to a specific input image highlights the fragments in the image that initially gave rise to the unit response^19,36^. Activation maximization takes the approach of iteratively deconvolving (300 iterations) the receptive field and adding it to the input image, to achieve an alternated image which elicits a maximal response of the model unit. The resultant putative receptive fields, presented in Fig. 4, appeared to share common aspects: they highlighted consistent fragments embedded in a larger part of the face image, with the right ear and left eye highlighted in the examples of panels a and b, respectively (red arrows). Nevertheless, there was a difference between the two methods, with the activation maximization revealing changes involving larger expanses of the units’ receptive fields.

### Match of neural to DCNN face-space in distinct ROIs

**Fig. 5:**
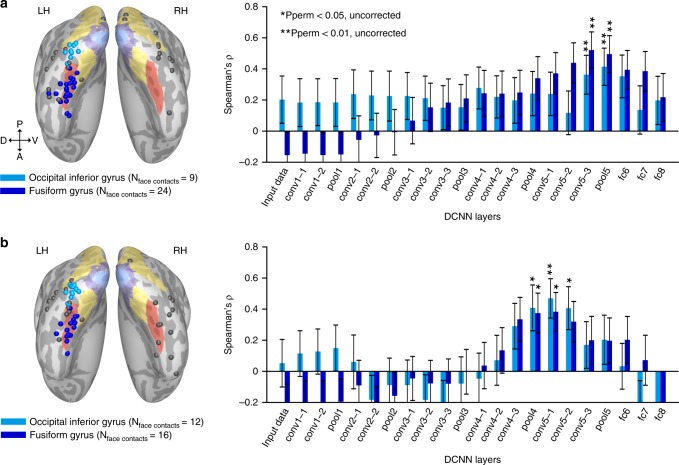
Match between neural and DCNN face-spaces in the occipital and temporal clusters of face contacts. **a** The same analysis as presented in Fig. 2a for set 1, with the difference of computing the correlation coefficients separately, for an occipital cluster (occipital inferior gyrus) and for a temporal cluster (fusiform gyrus) of face contacts. Anatomical locations of contacts assigned to the two clusters are presented on the inflated common cortical surface (surface colored labels are identical to those in Fig. 1a). Gray contacts were not assigned to any cluster. Error bars denote bootstrap s.e.m. on image pairs. **b** Same as panel **a** for the face contacts and images included in set 2. Anatomical labels were taken from individual patients’ cortical surface reconstruction; therefore, the contacts do not always fall within the targeted gyri on the common surface. Note that the correlation profile remained highly similar for the two clusters, with no significant difference found in any of the layers (permutation test on contacts’ ROI label)

Two prominent clusters of face contacts could be discerned anatomically in the left hemisphere (Fig. 1b, Supplementary Fig. 1)—an occipital cluster in the inferior occipital gyrus, likely corresponding to the occipital face area (OFA) and a temporal cluster in the fusiform gyrus, likely corresponding to the fusiform face area (FFA, composed of a posterior and an anterior patch in itself—pFus-faces/FFA-1 and mFus-faces/FFA-2^3^). Given the ongoing debate concerning the functional distinction between the two face patches, we separately examined their match to the DCNN layers. We defined the two clusters of face contacts based on the inferior occipital gyrus and the fusiform gyrus as anatomical regions of interest, and computed the neural to DCNN correlation for each cluster separately. This was performed only for set 1 and set 2, since set 3 did not include a sufficient number of contacts to conduct such an ROI analysis. As presented in Fig. 5,we found that for both sets, both clusters independently replicated the same correlation profile that we initially observed for the complete ensemble, with no significant difference in layer-selectivity arising when comparing the two clusters directly (contacts’ labels permutation test on correlation difference between the two clusters, all uncorrected *p* values for five intermediate layers >0.46).

Finally, we tested whether the observed DCNN to neural match was confined to the HFA signal or whether it could be revealed also in the LFP evoked responses. To this end, we recomputed the neural match to DCNN layers, this time extracting the mean evoked response from the raw (common referenced) iEEG signal. The analysis failed to reveal a significant correlation to any of the DCNN layers, in any of the three sets. Similarly, trial by trial extraction of the instantaneous band limited power at 8–13 Hz failed to reveal a significant correlation with the DCNN face-space in any of the layers or sets.

## Discussion

The present findings reveal a significant match between the face-space geometry of human face-selective neuronal groups recorded intracranially and a DCNN (VGG-Face) capable of human-level face recognition performance. The match was absent for the same network when untrained and assigned with random connection weights. In addition, the match was significant in the same range of mid-layers both when pooling all electrodes across patients and when averaging individual matching profiles of patients with a minimum of five face contacts (Supplementary Fig. 5). These results point to an intriguing convergent evolution of face-space geometry, as reflected in face-pair pattern similarities emerging both in biological and in artificial networks^20^. This convergence highlights the functional importance of activation pattern similarity to face perception and thus extends earlier theoretical proposals by Edelman and Grill-Spector^25^ of the fundamental role of such similarities in object representations as well as more recent ones in the domain of object categories^22^. The results are also compatible with our previous study, showing significant correlation between face exemplar activation distance measures and their perceptual similarity^37^ (but see^38^).

A number of previous studies have employed ad-hoc models and classifiers to successfully decode face exemplars from brain data (for a recent example see e.g.^39^). However, these models, applied to specific experimental data sets, have not been demonstrated to perform at the level of realistic human recognition capacity. The added value of DCNNs is that they are trained independently of neural data and, critically, reach human-level performance. As such, they offer novel opportunities to highlight characteristics of the neural representations that are essential to performing the recognition tasks. Thus, detecting commonalities between representational principles found in high performing models such as DCNNs and large-scale neural data provides valuable pointers to features of the neural representations essential for reaching high levels of task performance.

It is important to emphasize that face perception is a multi-faceted process that involves, on the one hand, a pictorial function, i.e. our essentially limitless ability to distinguish among different images of faces and, on the other hand, a recognition function, in which we can identify specific personal identities across a diverse set of different view-points and appearances. At present, there is ample evidence pointing to the involvement of face-selective areas in mid-high visual cortex in face perception and recognition—including fMRI (e.g.^40^), clinical^5^ and stimulation^7,8,10,41^ data. However, given the complex hierarchical nature of processing in the human ventral stream, it is difficult to derive from stimulation and clinical data the precise role of neuronal groups in the processing cascade and the integration leading to the overall human perceptual capabilities.

Three converging lines of results point to a pictorial function of the human face selective neuronal groups in the present study. First, the face-space geometry match was consistently restricted to the mid-hierarchical layers of the DCNN, arguing both against strictly low-level feature representations at the bottom end and viewpoint invariant representations at the high end (Fig. 2). This could not be attributed to a difference between the occipital and temporal clusters since separately computing the neural to DCNN match for the inferior occipital gyrus (OFA anatomical marker) and fusiform gyrus (FFA anatomical marker) recording sites failed to reveal a shift towards higher DCNN layers for the temporal fusiform cluster (Fig. 5). Indeed, examining the viewpoint sensitivity across the different layers of the VGG-Face network (Supplementary Fig. 3) revealed that layers showing the highest match to the brain responses were sensitive to view point manipulations, further confirming their pictorial rather than identity-preserving nature.

The neural to DCNN correlation was never restricted to a specific layer and the significant correlations were slightly jittered in hierarchical level across the three sets of images and face contacts, ranging from layer pool4 to layer pool5. The source of this variability is not clear at present. One possibility is that this cross-layer blurring was due to the limited sampling density of the iEEG contacts that necessitated pooling across face-selective sub-regions in visual cortex^3^, which may be differentially correlated to the DCNN layers.

The second line of evidence concerns image manipulations targeting low-level features, such as removing all background information, changing luminance or converting the face colors to gray. Applying such manipulations to the DCNN input images had no significant effect on the neural to DCNN match (Fig. 3, Supplementary Fig. 9) or the DCNN representations in the brain-matching layers (Supplementary Fig. 11). By contrast, higher level manipulations of pictorial aspects of the faces which nevertheless preserved their personal identity (Fig. 3) showed a significant reduction in the neural to DCNN match and in the match between the DCNN representations themselves (see Supplementary Fig. 11a), pointing to the dependency of the effect on some high-level pictorial aspects rather than on view-invariant personal identity. This result is compatible with previous studies that demonstrated viewpoint selectivity in the activation patterns of face-selective regions^42–45^. It should be emphasized, however, that our results pertain to the level of invariance in the DCNN layers alone, and that without collecting neural data following image manipulations we cannot rule out the possibility that the neural face-space may remain invariant to such high-level identity-preserving image manipulations.

Finally, visualizing the receptive fields of artificial units whose selectivity profiles matched those of specific face contacts revealed consistent viewpoint selective face fragments such as ears and eyes. These fragment-like receptive fields are compatible with previous reports of the selective activation of face areas to informative fragments^28^. Interestingly, the two different approaches we applied to reveal these receptive fields highlighted different levels of holistic representations, with a more a localized receptive field revealed through the deconvolution method while a more gestalt-like receptive field revealed through the activation maximization approach (Fig. 4). This dependency of the receptive field properties on their mapping method is reminiscent of the finding of a more localized RF in primate infero-temporal cortex when a reductive method is applied^46^ vs. more holistic RF properties that were revealed when using face-template approaches (e.g. refs. ^12,47^).

An interesting question is whether the match between brain and DCNN face-spaces was specific to a network trained to identify faces. We explored this question by comparing the correlations found with the VGG-Face to those obtained for a network having identical architecture, but trained on ImageNet for a more general object-categorization task (VGG-16). Interestingly, our results showed that a similar correlational pattern emerged for both VGG-Face and VGG-16, suggesting that training on the specific task of face identification is not a necessary prerequisite for the match to emerge. This result points to the possibility that the features underlying the match to the neural face-space in VGG-16 are not face-specific, but rather generic object features which can be recomposed to construct a representational space with a similar geometry to the neural face-space.

A limitation to note is that a greater proportion of face contacts in the present study were localized in the left hemisphere (44/58, 41/53 and 13/23 in set 1 to 3, respectively), with the ROI analysis (Fig. 5) focused solely on left hemisphere face contacts. Given previous evidence suggesting that face perception in humans is dominated by the right hemisphere^5,48^ (but see e.g.^6^), this may have biased our match towards more mid-levels DCNN layers.

Overall, our results suggest a functional role for human face-selective neuronal groups in representing how face images look. Our results are thus compatible with the notions that person identity representations that show invariance to face appearance, such as viewpoint changes, may be found in more downstream medial temporal lobe structures^49^ or by the extended nodes of the face network in anterior temporal and frontal cortices that we failed to sample^50,51,45^. A cautionary note is in order here since the present results were obtained when the patients performed a 1-back memory task and not an explicit recognition task, with most faces being unfamiliar. At this point we cannot rule out the possibility that under a person identification task the correlation may shift to higher, more invariant, layers of the VGG-Face network. This possibility will actually be an intriguing extension of the functional importance of similarity distances in face perception. On the other hand, it is important to note that previous studies attempting to reveal increased invariance to familiar faces in ventral stream face regions showed contradictory evidence^42,52,53^. Furthermore, it should be noted that previous studies showing representational similarities across categories between DCNNs and non-human/human primates used passive viewing^19,20,22^ or an oddball variant^23,24^ as the main task.

In summary, the revolutionary discovery that artificial deep convolutional networks can achieve human-level recognition performance offers, for the first time, a truly realistic model of high order visual processing. Together with the large scale iEEG recordings of visual neuronal groups in humans, they allowed fresh insights into the functional role and mechanistic generation of highorder human face representations. Employing the rapidly evolving new DCNNs may help in the future to resolve outstanding issues such as the functionalities of distinct cortical patches in high order visual areas and the role of top down and local recurrent processing in brain function^54^.

## Methods

### Participants

Sixty-one participants monitored for pre-surgical evaluation of epileptic foci were included in the study, 33 of them had face selective contacts (11 females, mean age 35 years, SD = 11.6; see Supplementary Table 1 for individual demographic, clinical, and experimental details). All participants gave fully informed consent, including consent to publish, according to NIH guidelines, as monitored by the institutional review board at the Feinstein Institute for Medical Research, in accordance with the Declaration of Helsinki.

### Tasks and stimuli

Three versions of a 1-back visual task were included in this study, each consisting a different set of 10 face images as well as a different set of additional images from other categories (see Fig. 1a for a schematic illustration of the three tasks). Each patient performed either one or two versions out of the three (for individual specification see Supplementary Table 1). Face stimuli in task 1 (set 1) were natural face images of famous people collected in an internet search. This task version consisted of a total of 60 stimuli: 10 faces, animals, tools, words, patterns, and places. Images were presented for 250 ms and were followed by a jittered inter stimulus interval ranging from 750 to 1050 ms. The task included 360 trials, 24 of which were 1-back repetitions. Each image exemplar was presented six times throughout the task. Face stimuli in task 2 (set 2) were taken from a database previously reported^55^. This task version consisted of a total of 56 stimuli, including in addition to the 10 face stimuli also images of tools, patterns, houses, and body parts. Images were presented for 250 ms at a fixed pace of 1 Hz. The task included 205 trials, 25 of which were 1-back repeats. Each image exemplar was presented 3–4 times throughout the task. Face stimuli for task 3 (set 3) were taken from an open-source database^56^. This task version consisted of a total of 50 stimuli: 10 faces, tools, patterns, houses, and body parts. This was a block-design task, with each block consisting 10 images from the same category, presented in pseudo-random order (each exemplar presented once during each block, aside from the case of 1-back repeats). Images were presented for 500 ms, followed by a jittered inter stimulus interval ranging from 750 to 1500 ms. Blocks were separated by either 4 or 8 s. The task included 260 trials (26 blocks), 18 of which were 1-back repeats. Each image exemplar was presented 4–5 times throughout the task. In all three versions, stimuli were squared and centrally presented, subtending a visual angle of approximately 13° in task 1 and 11° in tasks 2 and 3. During the tasks, participants were seated in bed in front of an LCD monitor. They were instructed to maintain fixation throughout the task and to click the mouse button whenever a consecutive repetition of the exact same image occurred. Five of the participants (one in set 1, two in set 2, and two in set 3) were instructed to press on each trial, not only on 1-back repeats, to indicate whether a 1-back repeat occurred or not.

### Electrodes implant and data acquisition

Recordings were conducted at North Shore University Hospital, Manhasset, NY, USA. Electrodes were either subdural grids/strips placed directly on the cortical surface and/or depth electrodes (Ad-Tech Medical Instrument, Racine, Wisconsin, and PMT Corporation, Chanhassen, Minnesota). Subdural contacts were 3 mm in diameter and 1 cm spaced, whereas depth contacts were 2 or 1 mm in diameter and 2.5 or 5 mm spaced, for Ad-Tech and PMT, respectively. Maximal depth of depth electrodes was 70 mm, corresponding to a maximum of 13 implanted contacts. The signals were referenced to a vertex screw or a subdermal electrode, filtered electronically (analog bandpass filter with half-power boundaries at 0.07 and 40% of sampling rate), sampled at a rate of either 512 or 500 Hz and stored for offline analysis by XLTEK EMU128FS or NeuroLink IP 256 systems (Natus Medical Inc., San Carlos, CA). Electrical pulses were sent upon stimuli onsets and recorded along with the iEEG data for precise alignment of task protocol to neural activity.

### Anatomical localization of electrodes

Prior to electrodes implant, patients were scanned with a T1-weighted 0.8 mm isometric anatomical MRI on a 3 Tesla Signa HDx scanner (GE Healthcare, Chicago, Illinois). Following the implant, a computed tomography (CT) and a T1-weighted anatomical MRI scan on a 1.5 Tesla Signa Excite scanner (GE Healthcare) were collected to enable electrode localization. The post-implant CT was first aligned with the post implant-MRI and then with the pre-implant MRI using a rigid affine transformation as implemented by FSL’s Flirt^57^. This allowed visualization of the post-implant CT scan on top of the pre-implant MRI scan. Individual contacts were then identified manually by inspection of the CT on top of the pre-implant MRI and were marked in each patient’s pre-implant MRI native space, using BioImage Suite^58^.

Electrode projection onto the cortical surface was performed as previously reported^59^ (see also^60^ for an open source tool box with a similar pipeline). Individual patients’ cortical surface was segmented and reconstructed from the pre-implant MRI using FreeSurfer 5.3^61^, and each electrode was allocated to the nearest vertex on the cortical surface. To project electrodes from all patients onto a single template, the unfolded spherical mesh of each individual was resampled into a standard unfolded spherical mesh using SUMA^62^. Colored labels on the cortical surface as presented in Fig. 1b were derived from surface-based atlases as implemented in FreeSurfer 5.3: functional atlas of retinotopic areas^63^ (intermediate retinotopic areas); Destrieux anatomical atlas^64^ (Fusiform gyrus); and Juelich histological atlas (V1 and V2 corresponding to Brodmann areas 17 and 18, respectively).

### iEEG signal preprocessing and HFA estimation

Signals that were initially recorded at a sampling rate of 512 Hz were down sampled to 500 Hz for consistency. Raw time-courses and power spectra of all channels were manually inspected for noticeable abnormal signals and other contaminations, and channels appearing as highly irregular were excluded from further analysis. Next, channels were re-referenced by subtracting the common average signal from the intact channels.

To estimate high-frequency amplitude (HFA) modulations, the signal was divided into nine frequency sub-ranges of 10 Hz width, ranging from 48 to 154 Hz. The sub-ranges did not include 59–61 and 117–121 Hz to discard line noise. The signal was band-passed at each frequency sub-range and instantaneous amplitude in each sub-range was estimated by taking the absolute value of the filtered signal’s Hilbert transform^65^. Since the 1/*f* profile of the signal’s power spectrum results in greater contribution of lower frequencies to the overall HFA estimation, we normalized each sub-range by dividing it with its mean value, and averaged the normalized values across all nine sub-ranges. All data preprocessing and analyses were carried out using in house Matlab codes (R2017a). For filtering of frequency sub-ranges, we used original EEGLAB’s Hamming windowed FIR filter (pop_eegfiltnew function^66^).

### Definition criteria of face selective contacts

Face selective contacts were defined as visually responsive contacts with a significant response to faces compared to places and compared to patterns: First, we tested whether the mean response  of each contact to all available stimuli from the versions the patient participated in were significantly greater than baseline (paired *t*-test on mean exemplar responses versus baseline, at 50–500 ms and −200 to 0 ms relative to image onset, respectively). Although images were presented for different durations across the task versions (250 ms in sets 1–2 and 500 ms in set 3), we pre-defined the response window to be identical across the three sets. Hit, miss, and false alarm trials were excluded from all analyses. FDR correction was then applied to the pooled *p* values from all 61 patients. Contacts with pFDR < 0.05 and a considerable effect size (Glass’ Δ) of larger than 1 were defined as visually responsive. Six hundred and forty-two out of 8916 contacts were found to be visually responsive. Next, visual contacts that were significantly more selective to faces when contrasted with places and when contrasted with patterns were defined as face contacts (two Wilcoxon rank sum tests per contact, *p* < 0.05, uncorrected). Finally, we applied anatomical constraints whereby face contacts located within V1, V2, or in frontal regions, as well as contacts localized further than 10 mm from the cortical surface, were excluded. This resulted in the final set of 96 face contacts from 33 patients: 58, 53, and 23 face contacts in set 1, 2, and 3, respectively. Thirty-four contacts (10 patients) overlapped between sets 1 and 2, and 4 contacts (2 patients) overlapped between sets 1 and 3. The cortical distribution of face contacts is presented separately for each set in Supplementary Fig. 1. None of the detected face contacts were identified as located over the seizure onset zones by an epileptologist’s inspection.

### Exemplar selectivity index of individual face contacts

To assess the level of selectivity to different faces within each face-selective contact we defined a face exemplar selectivity index as the *d*′ between the most- and least-preferred face images:

1 $${\mathrm{face}}\;{\mathrm{exemplar}}\;{\mathrm{selectivity}}\;{\mathrm{index}} = \frac{\mu _{\mathrm{best}} - \mu _{\mathrm{worst}}}{{\sqrt {\frac{1}{2}\left( {\sigma _{\mathrm{best}}^2 + \sigma _{\mathrm{worst}}^2} \right)} }},$$

where *μ*_Best/Worst_ denotes the average of mean HFA responses at 50–500 ms across all repetitions, and $\sigma _{{\mathrm{best}}/{\mathrm{worst}}}^2$ denotes the variance across repetitions of the relevant face exemplar. Selectivity indices presented in Fig. 1b were computed on all task versions available to the specific face contact. If a face contact was included in more than 1 task version (maximal versions per contacts are two), indices were averaged across the versions. Selectivity indices presented in Supplementary Fig. 1 were computed on face images included in the specific set presented in each of the three panels. Statistical significance in each face contact was assessed by a permutation test, in which the same index was computed for 1,000 random shuffles of single trials labels. *P* values were defined as the proportion of shuffle-derived indices that exceeded the original index.

### Decoding face exemplars from face contacts ensemble activity

To decode specific face exemplars from the activation pattern of all face contacts we applied a simple template matching decoding scheme in each of the three sets (see Supplementary Fig. 2a for a schematic illustration of the decoding procedure): First, data were ordered in a grand three-dimensional data matrix, **G**, of the form ${\mathbf{G}}_{{\mathrm{face}}\;{\mathrm{exemplars}}\;{\times}\;{\mathrm{face}}\;{\mathrm{contacts}}\;{\times}\;{\mathrm{trials}}}$. Entry *i*,*j*,*k* in this matrix is the response to the *i*th face exemplar in the *j*th face contact, in the *k*th repetition trial, smoothed with 50 ms running average window. Importantly, this response is a time series of 450 ms, taken at 50–500 ms relative to image onset. Second, we randomly chose a single trial from each face contact to each face exemplar and assigned it to a test pattern matrix, **T**, of the form ${\mathbf{T}}_{{\mathrm{face}}\;{\mathrm{exemplars}}\;{\times}\;{\mathrm{face}}\;{\mathrm{contacts}}}$. The remaining trials in the grand three-dimensional data matrix were averaged and assigned to a reference matrix, **R**, of the form ${\mathbf{R}}_{{\mathrm{face}}\;{\mathrm{contacts}} \times {\mathrm{face}}\;{\mathrm{exemplars}}}$. Thus, every decoding iteration began with a test and a reference two-dimensional matrices (**T**and **R**, respectively): row *i* in the test matrix is the concatenated vector of randomly chosen single trial responses from all face contacts to the ***i***th face exemplar, whereas row *i* in the reference matrix is the concatenated vector of averaged responses across the remaining trials from all face contacts to the *i*th face exemplar. Third, we assigned 10 decoded labels based on the minimal Euclidean distance between each row in the test and reference matrices: on every decoding step, the minimal distance between any row of the test matrix and any row of the reference matrix was detected, and the label of the row in the reference matrix (e.g. Face 1) was assigned to its minimally distant row in the test matrix. The detected pair of test-reference rows was then excluded from the subsequent decoding steps such that each reference row could only be assigned once in every decoding iteration. One thousand decoding iterations were performed. The mean percentage of accurately decoded face exemplars across the 1000 iterations was defined as the decoding accuracy.

### Pair-wise distances in the neural face-space

Pair-wise distances were computed on the neural face representation matrix, which is the same grand data three-dimensional matrix described in the previous section (and illustrated in Supplementary Fig. 2a), after averaging across the trials dimension to receive a two-dimensional matrix of the form ${\mathrm{face}}\;{\mathrm{exemplars}} \times {\mathrm{face}}\;{\mathrm{contacts}}$. Pair-wise distance between the neural responses to two exemplars was then computed as the Euclidean distance between the two rows in the matrix that correspond to the pair of exemplars. Forty-five distance values were extracted per set, following the equation $N\ast (N - 1)/2$, with *N* denoting the number of face exemplars (*N* = 10 in all 3 sets).

To test the contribution of the exact wave form during the 450 ms HFA response to the DCNN to brain match (Supplementary Fig. 7), we also averaged the mean HFA response in each entry of the neural face representation matrix across time, resulting in a single mean amplitude value instead of a 450 ms response time series. All subsequent analyses addressing the match to DCNNs were otherwise identical.

To investigate the specificity of the DCNN to brain match to the HFA signal, we computed the neural pair-wise distances for two additional signals, focusing on low frequencies of the iEEG signal: the conventional ERP and the instantaneous power of low frequencies at 8–13 Hz. ERP responses were extracted by locking the common-referenced raw signal to image onsets and averaging across repetitions of the same exemplar. Low frequencies band limited power was computed by filtering the common-referenced signal at 8–13 Hz and extracting the absolute value of the Hilbert transformed signal. The response window for both ERP and low-frequency instantaneous power was pre-defined at 125–250 ms relative to image onset.

### DCNN models

We used the pre-trained VGG-Face feedforward architecture of Parkhi et al.^17^. The network consists of five stacked blocks followed by three fully connected (fc) layers. Each block consists of 2–3 consecutive convolutional layers (13 convolutional layers in total) followed by max pooling. All 13 convolutional layers and 3 fully connected layers are followed by a rectification linear unit (ReLU), introducing non-linearity to the model. The network was trained on a large scale data set with over two million face images for recognition of 2622 identities. This model reached nearly perfect performance, comparable to state of the art models (e.g. DeepFace^16^). Importantly, none of the identities presented in our three tasks were included in the set used to train the network. Preprocessing of our raw stimulus images prior to passing them forward through the network included resizing to match network input size of 224 × 224 pixels and mean RGB channel value subtraction, as estimated from the network’s training set.

To compute DCNN pair-wise distances, we passed forward the face images through the network and extracted the activations of all units at each layer. We then computed the Euclidean distance between pairs of activation patterns corresponding to image pairs, to generate a pair-wise distances vector at each layer.

We also tested a DCNN trained on object classification—VGG-16^34^. VGG-16 has an identical architecture to VGG-Face, yet it differs in that the network, initialized at random weights, was trained on ImageNet to recognize 1000 possible object classes.

### Estimation of similarity between DCNN and neural face-spaces

The neural pair-wise distances vector was correlated with the 22 DCNN pair-wise distances vectors derived from each layer using Spearman’s correlation. To assess statistical significance of the correlation to each layer, we shuffled image labels in the neural data 1000 times and recomputed the neural distances vector while leaving the DCNN distances vectors fixed. *P* value assigned to each layer was the proportion of correlation values derived from shuffled data that exceeded the original correlation value. An FDR correction was then applied on the resultant 22 *p* values to control for a 5% false discovery rate across the layers.

To estimate the consistency in correlation patterns across the three sets (Fig. 2d), we computed the weighted average of correlation coefficients with each DCNN layer across the three sets. Following^67^, the weighted correlation ($\hat R_z$) and standard error (*SE*_*z*_)in every layer was defined as

2 $$\hat R_z = \frac{{\mathop {\sum }\nolimits_{\mathrm{i}} R_{\mathrm{z}}^{\mathrm{i}}\;n^{\mathrm{i}}}}{{\mathop {\sum }\nolimits_{\mathrm{i}} n^{\mathrm{i}}}},$$

3 $${\mathrm{{SE}}}_{\mathrm{z}} = \frac{{\mathop {\sum }\nolimits_{\mathrm{i}} (R_z^i - \hat R_z)^2n^{\mathrm{i}}}}{{\sqrt N \mathop {\sum }\nolimits_{\mathrm{i}} n^{\mathrm{i}}}},$$

where $R_z^i$ is the fisher *z* transformed Spearman’s correlation in set *i*; *n*^*i*^ is the number of face contacts in set *i*; and *N* is the total number of sets. The resultant $\hat R_z$ and SE_*z*_ were then back transformed. Computing the non-weighted means gave highly similar results, with the range of significant layers unchanged.

### Controlling for variations in low-level image parameters

To account for the potential impact of pair-wise distances between low-level image parameters on the DCNN to brain match, we computed the partial correlation between the neural and DCNN pair-wise distances, partialing out the contibution of pair-wise distances in luminance, RMS contrast, gradient, and saturation to the observed match. These four parameters were computed as in a recent study^68^:

4 $${\mathrm{Luminance}}: < {\mathrm{gray}}\;{\mathrm{scale}}\;{\mathrm{pixels}} > ,$$

5 $${\mathrm{RMS}}\;{\mathrm{contrast}}:{\mathrm{std}}({\mathrm{gray}}\;{\mathrm{scale}}\;{\mathrm{pixels}}),$$

6 $${\mathrm{Gradient}}:\mathop {\sum }\limits_{{\mathrm{gray}}\;{\mathrm{scale}}\;{\mathrm{pixels}}} \sqrt {(\nabla {\mathrm{horizontal}}^2 + \nabla {\mathrm{vertical}}^2)},$$

7 $${\mathrm{Saturation}}:\frac{1}{{N_{{\mathrm{pixels}}}}}\mathop {\sum }\limits_{{\mathrm{pixels}}} \frac{{\max \left( {{\mathrm{R}},{\mathrm{G}}.{\mathrm{B}}} \right) - {\mathrm{min}}({\mathrm{R}},{\mathrm{G}},{\mathrm{B}})}}{{\max \left( {{\mathrm{R}},{\mathrm{G}}.{\mathrm{B}}} \right)}},$$

Where RGB to gray scale conversion follows the transformation:

8 $${\mathrm{Gray}}\;{\mathrm{scale}}\;{\mathrm{value}} = 0.299{\mathrm{R}} + 0.587{\mathrm{G}} + 0.114{\mathrm{B}}.$$

Note that for set 2, which included only gray scale images, parameters were computed on the original pixel values, without first converting them into gray scale. In addition, the saturation parameter (Eq. (7)) is not applicable to gray scale images and was therefore not extracted or analyzed for set 2.

### Low- and high-level image manipulations

Background removal of face images were carried out using Adobe Photoshop. Luminance matching was performed independently per set, by computing the mean luminance of all exemplars in the set (Eq. (4)), and adding to all pixels of each image the difference value between its luminance and the average luminance across images. For colored sets (set 1 and set 3), this operation was carried on the value plane in the HSV format, followed by conversion back into RGB format.

For high-level face manipulations, including different appearance and two-stage view shifts we took advantage of the faces in set 1 depicting famous people and searched for available images of the same identities taken from a half profile and a full profile (Fig. 3, light blue and dark blue bars), and from a similar view but under a different appearance (e.g., facial expression, haircut). All manipulated faces were resized and aligned to optimally overlap with their original counterparts.

To assess statistical significance of the impact of the above mentioned six manipulations on the DCNN to brain match and the DCNN to DCNN match, we applied a permutation test per manipulation and layer. Neural distances were recomputed following 1000 image permutations. *P* value assigned to each layer and manipulation was the proportion of correlations deltas given shuffled labels that exceeded the original change in correlation. In addition, the impact of manipulations on the DCNN to brain match was also tested using a 95% image bootstrap confidence interval around the original correlation value, resulting in the same significance profile across manipulations.

### Identity decoding from DCNN layers

To assess the DCNN capability in generalizing identity across high-level identity-preserving manipulations, we pooled the images from the three high-level manipulations presented in Fig. 3a (different appearance, ~45° rotation and ~90° rotation) and the original images to one data set. We then randomly assigned one image of each identity to constitute a ten images test set, and applied a Euclidean distance nearest neighbor classifier to classify the identities in the test set. We repeated this procedure 1000 times per layer, resulting in a mean classification accuracy for the two layers that matched the neural face-space in set 1 and for the top layer in the network, fc8 (Supplementary Fig. 10).

### Detection of DCNN model units

We attempted to find single artificial units (termed here model units) that can significantly predict the responses of single face-selective contacts to different face exemplars. We deployed the following search scheme per face contact in each of the three sets: first, the linear correlation between the neural responses (HFA amplitude at 50–500 ms, averaged across repetitions and time) and each of the layer’s units was computed for nine out of the ten faces. Next, we took the unit that best correlated to the nine neural responses and used a least-squares linear fit to predict the neural response to the held out tenth face. We repeated this procedure ten times, holding out a different face exemplar on every iteration. If the same unit was best correlated to the neural responses in all leave-one out folds, and resulted in a significant linear prediction of the 10 held out neural responses based on the linear fit (label permutation test, *p* < 0.05), the unit was defined as a model unit. Note that this search was carried out only in the DCNN layers that significantly matched the neural face-space (two layers in each set, see Fig. 2a–c). Finally, we applied a cluster correction to further ensure the received model units could not be detected by chance: we repeated the same search procedure 1000 times, each time shuffling image labels of the neural responses. This resulted in a distribution of 1000 model units counts detected given shuffled data. Only if the original number of detected model units in a given layer exceeded the 95th percentile of the count distribution derived from shuffled labels, we qualified the model units found in the layer as significant. For set 1, we found five and two model units in layer pool5 and layer conv5–3, respectively. For set 2, we found a single model unit that did not survive cluster correction.

### Receptive field visualization of model units

We used two techniques to visualize the receptive field of detected model units. The first technique is commonly referred to as deconvolution, established by Zeiler and Fergus^19,36^. Their method allows to feedforward an input image through a network, and then propagate it backwards to the image space given solely its representation in a unit of interest (i.e. setting all other units in the layer of the target unit to zero). The resultant back-propagated image thus unveils the receptive field of a defined target unit in the network as casted on the original input image—an approximation of the weighted fragment in the original image that elicited the target unit’s response. In practice, we based our implementation of the deconvolution technique on a publicly available TensorFlow implementation^69^.

The second technique that we used is commonly termed activation maximization^70^. Here, we sought to iteratively alter the input image in a way that, once fed into the network again, would maximally increase the response of a target model unit. In our implementation, we applied the decovolution technique iteratively (300 iterations): on each iteration we estimated the receptive field of the target unit using decovolution and added the deconvolved image, multiplied by a learning rate of 200, to the initial input image of that iteration. The resultant image was then used as the input image for the subsequent iteration. The visualizations presented in Fig. 4 and Supplementary Fig. 12 are the deltas between the RBG channels of the deconvolved image at the last iteration and the original image.

### Region of interest analysis

Two anatomical clusters in set 1 and in set 2 were defined (set 3 did not include a sufficient amount of contacts to perform an region of interest analysis). Face contacts assigned to the occipital and temporal clusters were defined in the individual cortical surface reconstruction as contacts located up to 3 mm from the left hemisphere inferior occipital gyrus and the left hemisphere fusiform gyrus, respectively. The subsequent analysis of the match between representational distances in the DCNN and in neural data (Fig. 5) was identical to that performed on the entire set of face contacts (Fig. 2).

### Reporting summary

Further information on research design is available in the Nature Research Reporting Summary linked to this article.

## Supplementary information

Supplementary Information

Reporting Summary

Source Data

## Data Availability

iEEG data and stimuli are available from the authors upon request. The VGG-Face model is available online at [http://www.robots.ox.ac.uk/~vgg/software/vgg_face/]. The source data underlying all main and supplementary figures are provided as Source Data files.

## Code availability

Matlab codes used for analyses and Python codes for receptive field visualization are available from the authors upon request.
