# Supplementary material for: Convergent evolution of face spaces across human face-selective neuronal groups and deep convolutional networks
Source: Nat Commun. 2019 Oct 30;10:4934. doi: 10.1038/s41467-019-12623-6 (PMC6821842; doi:10.1038/s41467-019-12623-6)
Supplement: Supplementary file 1 — Supplementary Information [file 41467_2019_12623_MOESM1_ESM.pdf]

nature research

Corresponding author(s): Rafael Malach

Last updated by author(s): Sep 12, 2019

Reporting Summary

Nature Research wishes to improve the reproducibility of the work that we publish. This form provides structure for consistency and transparency in reporting. For further information on Nature Research policies, see Authors & Referees and the Editorial Policy Checklist .

Statistics

For all statistical analyses, confirm that the following items are present in the figure legend, table legend, main text, or Methods section.

n/a	Confirmed	
☐	☒	The exact sample size (*n*) for each experimental group/condition, given as a discrete number and unit of measurement
☐	☒	A statement on whether measurements were taken from distinct samples or whether the same sample was measured repeatedly
☐	☒	The statistical test(s) used AND whether they are one- or two-sided<br>*Only common tests should be described solely by name; describe more complex techniques in the Methods section.*
☐	☒	A description of all covariates tested
☐	☒	A description of any assumptions or corrections, such as tests of normality and adjustment for multiple comparisons
☐	☒	A full description of the statistical parameters including central tendency (e.g. means) or other basic estimates (e.g. regression coefficient) AND variation (e.g. standard deviation) or associated estimates of uncertainty (e.g. confidence intervals)
☐	☒	For null hypothesis testing, the test statistic (e.g. *F*, *t*, *r*) with confidence intervals, effect sizes, degrees of freedom and *P* value noted<br>*Give P values as exact values whenever suitable.*
☒	☐	For Bayesian analysis, information on the choice of priors and Markov chain Monte Carlo settings
☒	☐	For hierarchical and complex designs, identification of the appropriate level for tests and full reporting of outcomes
☐	☒	Estimates of effect sizes (e.g. Cohen's *d*, Pearson's *r*), indicating how they were calculated

Our web collection on statistics for biologists contains articles on many of the points above.

Software and code

Policy information about availability of computer code

Data collection	Data was recorded and stored for offline analysis using XLTECEMU128FS or NeuroLink IP 256 systems.<br>Experimental tasks were programed in Presentation software (Neurobehavioral Systems)
Data analysis	Electrode localization and projection onto a common surface was performed using BioImage suit, FSL Flirt (CT to MRI alignment), Freesurfer (cortical surface reconstruction), SUMA (resampling to a common surface) and previously reported in-house Matlab code.<br>Signal processing and analyses were performed using custom Matlab codes (R2017a).<br>Receptive fields visualizations (Figure 4, deconvolution and activation maximization) were implemented<br>using adjusted open source codes in python.<br>DCNN activations extraction was performed both in Matlab (R2017a) and in Python (both yielded identical results)

For manuscripts utilizing custom algorithms or software that are central to the research but not yet described in published literature, software must be made available to editors/reviewers. We strongly encourage code deposition in a community repository (e.g. GitHub). See the Nature Research guidelines for submitting code & software for further information.

Data

Policy information about availability of data

All manuscripts must include a data availability statement. This statement should provide the following information, where applicable:

- Accession codes, unique identifiers, or web links for publicly available datasets
- A list of figures that have associated raw data
- A description of any restrictions on data availability

iEEG data and stimuli are available from the authors upon request.
The VGG-Facemodel is available online ([http://www.robots.ox.ac.uk/~vgg/software/vgg_face/]).
The source data underlying all main and supplementary figures are provided as Source Data files.

Field-specific reporting

Please select the one below that is the best fit for your research. If you are not sure, read the appropriate sections before making your selection.

- [x] Life sciences
- [ ] Behavioural & social sciences
- [ ] Ecological, evolutionary & environmental sciences

For a reference copy of the document with all sections, see nature.com/documents/nr-reporting-summary-flat.pdf

Life sciences study design

All studies must disclose on these points even when the disclosure is negative.

Sample size	Sample size was determined by the amount of intra-cranial recordings collected. 33 out of 61 patients that participated in 1-2 out of the 3 task versions were found to have 96 face selective contacts in total. 53 58 and 23 face contacts were available for set1, 2 and 3, respectively.
Data exclusions	Face contacts identified to be located over the seizure onset zone(s) were preset to be discarded from analysis. None of the face-selective contacts, however, were identified as such.
Replication	The reported main effect (i.e. correlation between neural face space and a DCNN face space) was replicated in 3 separate datasets, each consisting of a different set of face images and a different (however partially overlapping) set of face-selective intra-cranial contacts.
Randomization	Patients included in the analyses were pre-defined to have at least one face-selective contact.<br>As all analyses are pooled over patients' data, possible covariates are not relevant for the current design. An analysis aimed to assure the consistency of the reported main effect in individual patients was performed and a similar pattern to that obtained in the pooled analysis was observed (Figure S5).
Blinding	Data collection was preformed over the past 6 years in the North Shore University Hospital. Throughout data collection, the experimentalists who collected the data were entirely blind to the research question and planned analyses.

Reporting for specific materials, systems and methods

We require information from authors about some types of materials, experimental systems and methods used in many studies. Here, indicate whether each material, system or method listed is relevant to your study. If you are not sure if a list item applies to your research, read the appropriate section before selecting a response.

Materials & experimental systems

n/a	Involved in the study	
x		Antibodies
x		Eukaryotic cell lines
x		Palaeontology
x		Animals and other organisms
	x	Human research participants
x		Clinical data

Methods

n/a	Involved in the study	
x		ChIP-seq
x		Flow cytometry
	x	MRI-based neuroimaging

Human research participants

Policy information about studies involving human research participants

Population characteristics	Participants were patients monitored for pre-surgical evaluation of epileptic foci. 33 out of 61 participants were found to have face-selective contacts and were included in the analyses (11 females, mean age 35 years with SD=11.6).
Recruitment	All participants were diagnosed with intractable and drug-resistant epilepsy and were therefore administered to surgery in the North Shore hospital. Note that none of the face-selective contacts included in the analysis were located over epileptic foci.
Ethics oversight	Institutional review board at the Feinstein Institute for Medical Research

Note that full information on the approval of the study protocol must also be provided in the manuscript.

Magnetic resonance imaging

Experimental design

Design type	Prior to electrode implantation, patients were scanned with a T1-weighted 0.8 mm isometric anatomical MRI on a 3 Tesla Signa HDx scanner (GE Healthcare, Chicago, Illinois).<br>Following the implant, a computed tomography (CT) and a T1-weighted anatomical MRI scan on a 1.5

Tesla Signa Excite scanner (GE Healthcare) were collected to enable electrode localization.

Design specifications: n/a

Behavioral performance measures: n/a

Acquisition

Imaging type(s): structural

Field strength: 3 Tesla prior to implant and 1.5 Tesla post implant

Sequence & imaging parameters:
Pulse Sequence type: gradient echo
Imaging type: 3D GRE with IR preparation, Cartesian sampling
Field of view: 240mmX256mm
Matrix Size: 300X320
Slice Thickness : 0.8mm
Orientations : SAG
TE/TR/flip angle: 2400ms/2.22ms/8

Area of acquisition: whole brain

Diffusion MRI ☐ Used ☒ Not used

Preprocessing

Preprocessing software: Freesurfer for cortex segmentation and atlases labels.

Normalization: SUMA for resampling and projection onto a common surface template (std 141).

Normalization template: SUMA standard mesh template (std 141)

Noise and artifact removal: n/a for structural imaging

Volume censoring: n/a for structural imaging

Statistical modeling & inference

Model type and settings: n/a for structural imaging

Effect(s) tested: n/a for structural imaging

Specify type of analysis: ☐ Whole brain ☐ ROI-based ☐ Both

Statistic type for inference (See Eklund et al. 2016): n/a for structural imaging

Correction: n/a for structural imaging

Models & analysis

n/a	Involved in the study
☒	☐ Functional and/or effective connectivity
☒	☐ Graph analysis
☒	☐ Multivariate modeling or predictive analysis
