# Supplementary material for: Convergent evolution of face spaces across human face-selective neuronal groups and deep convolutional networks
Source: Nat Commun. 2019 Oct 30;10:4934. doi: 10.1038/s41467-019-12623-6 (PMC6821842; doi:10.1038/s41467-019-12623-6)
Supplement: Supplementary file 2 — Reporting Summary [file 41467_2019_12623_MOESM2_ESM.pdf]

Convergent evolution of face spaces across human face selective neuronal-groups and deep-convolutional networks

Supplementary Information

Grossman et al.

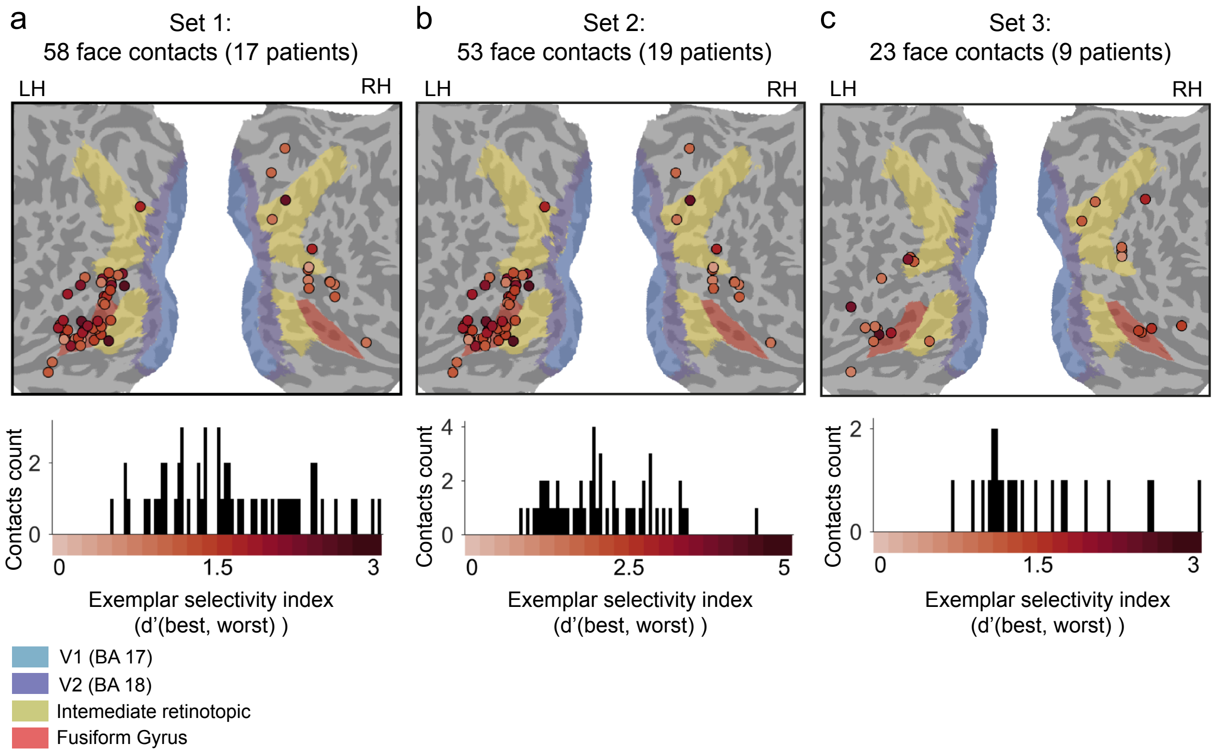

Supplementary Figure 1. Face contacts localizations and distribution of exemplar selectivity indices presented separately for the three sets. Panels depict the anatomical locations of face contacts included in sets 1-3 (panels **a-c**, respectively). Contacts are color coded according to their face exemplar selectivity indices as in Fig. 1b. By contrast to Fig. 1b, exemplar selectivity indices presented here were computed solely on the face images included in the relevant set.

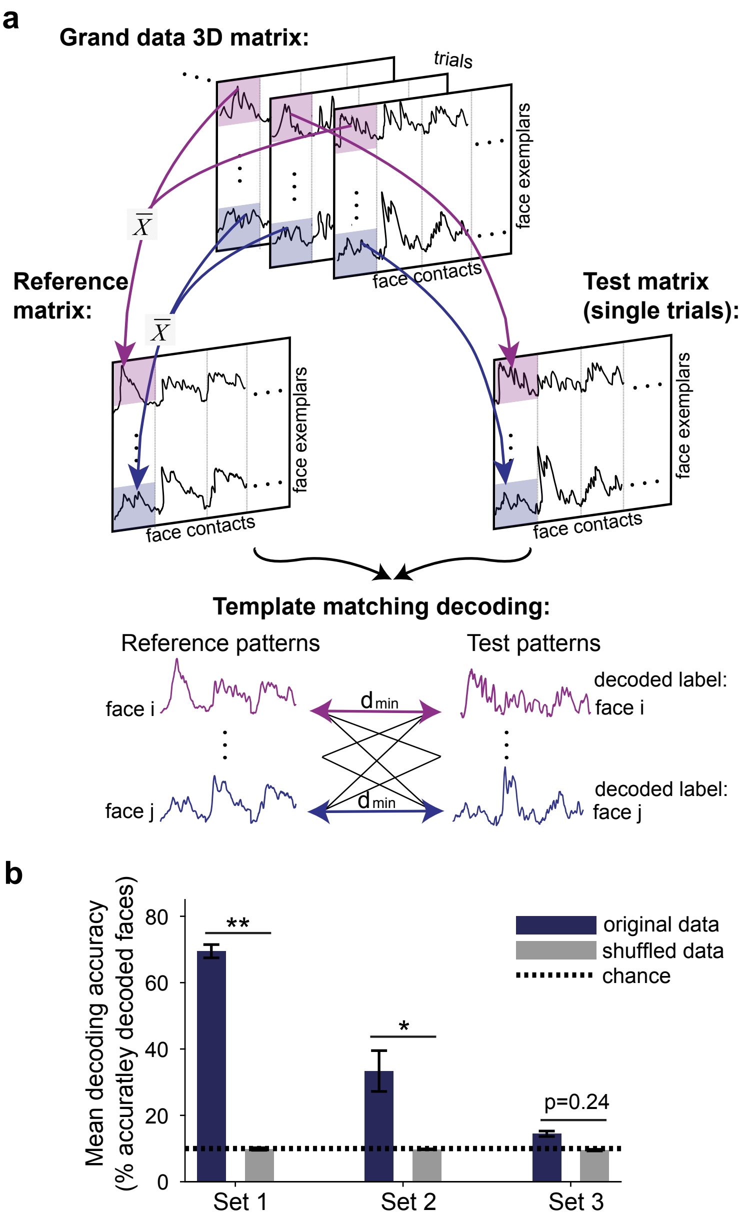

Supplementary Figure 2. Decoding face exemplars from activity pattern across face contacts. a Schematic illustration of a single decoding iteration. On every decoding iteration (N=1,000), a single response trial of each face contact to each exemplar was randomly assigned to a two dimensional test patterns matrix, while the remaining trials were averaged to constitute a two dimensional reference patterns matrix. Thus, each row in reference/test matrix is the concatenated responses pattern to a single face exemplar. Decoding was then performed based on the minimal Euclidean distances between rows in the test and in the reference matrices, assigning 10 decoded labels to the 10 rows of the test matrix. **b** Purple bars show mean decoding accuracy across 1,000 iterations, with error bars denoting the s.e.m. across face exemplars. Gray bars show mean decoding accuracies obtained from shuffling the test labels, which nicely fall on chance level (10%). P values per set were derived from an image permutation test, comparing the actual mean accuracy rate to the distribution of mean accuracy rates obtained from shuffled test labels (1,000 permutations).

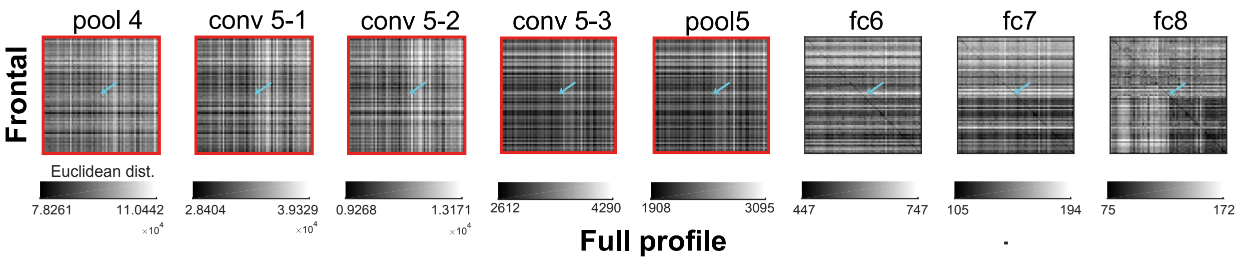

Supplementary Figure 3. Sensitivity of VGG-Face representations to view point changes in layers matching the neural face-space and in the subsequent three top fully connected layers. Each matrix presents the Euclidean distances between activations pairs for frontal and full right profile views of 65 identities. Diagonal therefore presents the distance between the same identities presented from a frontal and a profile view. Images were taken from a dataset (KDEF) independent of the ones used in the iEEG experiments. Red framed matrices are the intermediate layers, pool4 to pool5, that matched the neural face-space. Note the dominance of the diagonal (pointing to similar representations across view-points) emerging in the top fully connected layers, as compared to the layers matching the neural face-space.

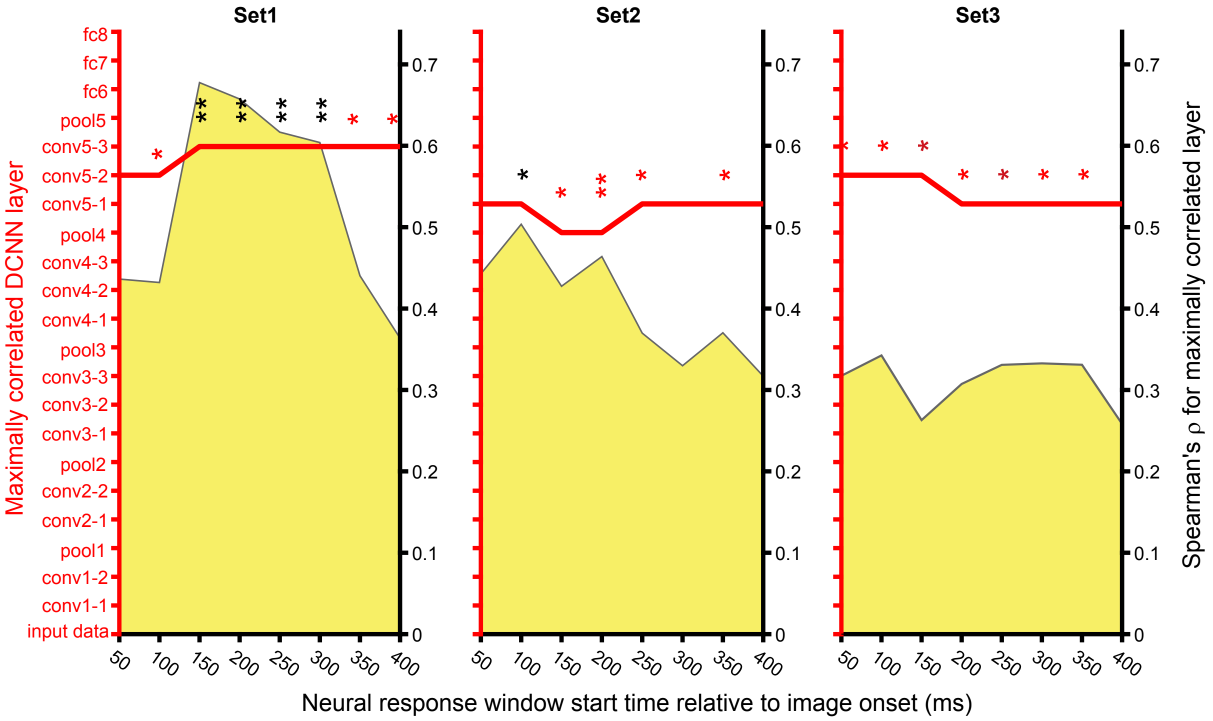

Supplementary Figure 4. Maximally correlated DCNN layer at different neural response times. The same RSA analysis between neural and DCNN layers face spaces as in Fig. 2 applied to different temporal segments of the neural responses, at a window of 200 ms with 50 ms stride between subsequent windows. The maximally correlated layer in VGG-Face at each time window is shown by the red trace. Corresponding correlation coefficients are depicted in yellow. Asterisks denote significance level at each time window, derived from an image permutation test and, when presented in black, followed by FDR correction across layers (red asterisks denote significance level prior to FDR correction). * $p<0.05$, ** $p<0.01$.

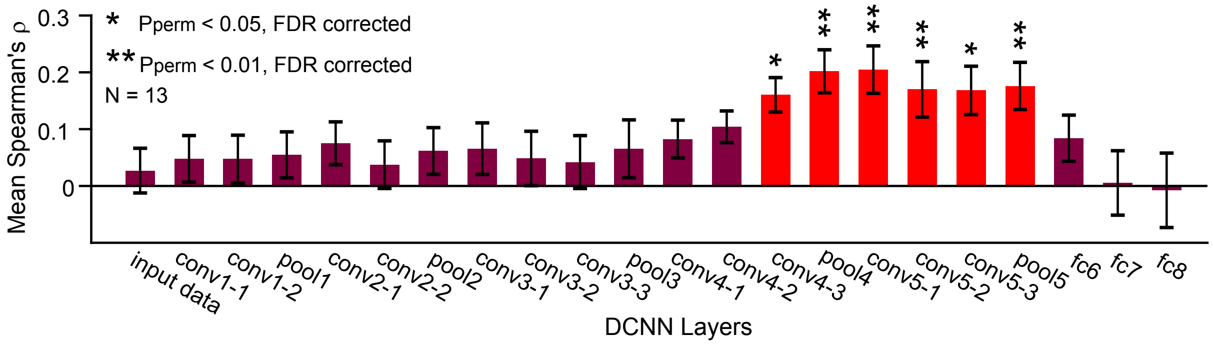

Supplementary Figure 5. Mean correlation between neural and DCNN face-spaces across individual patients with a minimum of 5 face contacts. The consistency of maximally correlated DCNN layers across individuals was tested by computing the neural to DCNN match in individual patients with a minimum of 5 face-contacts, and averaging the resultant Spearman's correlations. In total, 13 correlational patterns were computed (9 patients had a minimum of 5 face-contacts, 4 of them participated both in set 1 and in set 2). Bars' height denotes the average correlation across individual patterns (Fisher z transformation applied prior to averaging, and resultant mean values were transformed back). Error bars denote s.e.m. P values were derived from permuting image labels (1,000 permutations), followed by a 5% FDR correction for the number of layers.

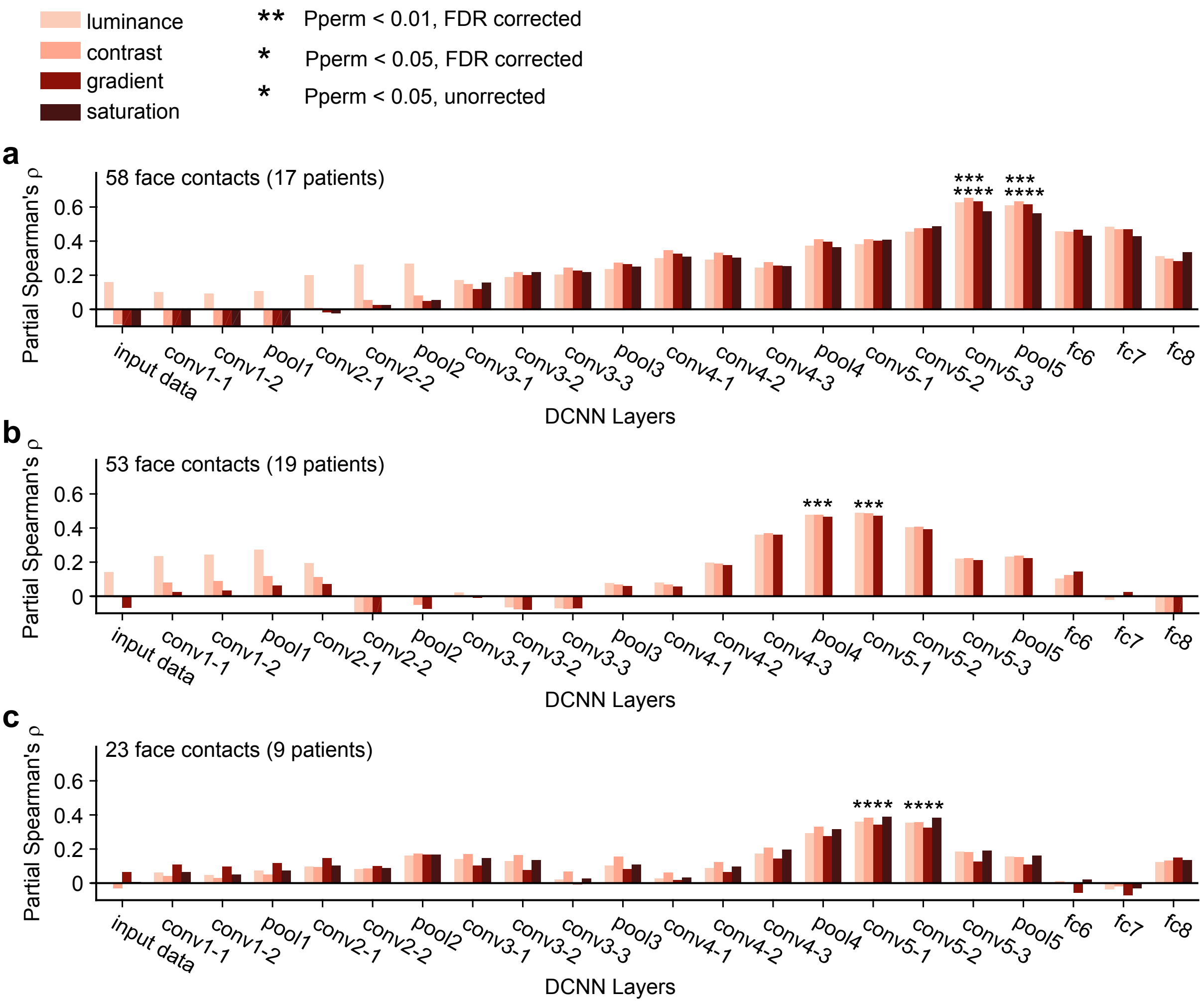

Supplementary Figure 6. Partial correlation of the DCNN to neural face-space match, controlling for pair-wise distances between low level image parameters. The same analysis as in Fig. 2a-c was repeated for sets 1-3 (panels **a-c**, respectively), this time applying partial correlation to partial out the possible contribution of pair-wise distances in luminance, RMS contrast, gradient and saturation. Note that the saturation is not applicable for gray scale images and was therefore not applied to set 2, which included only gray scale face exemplars.

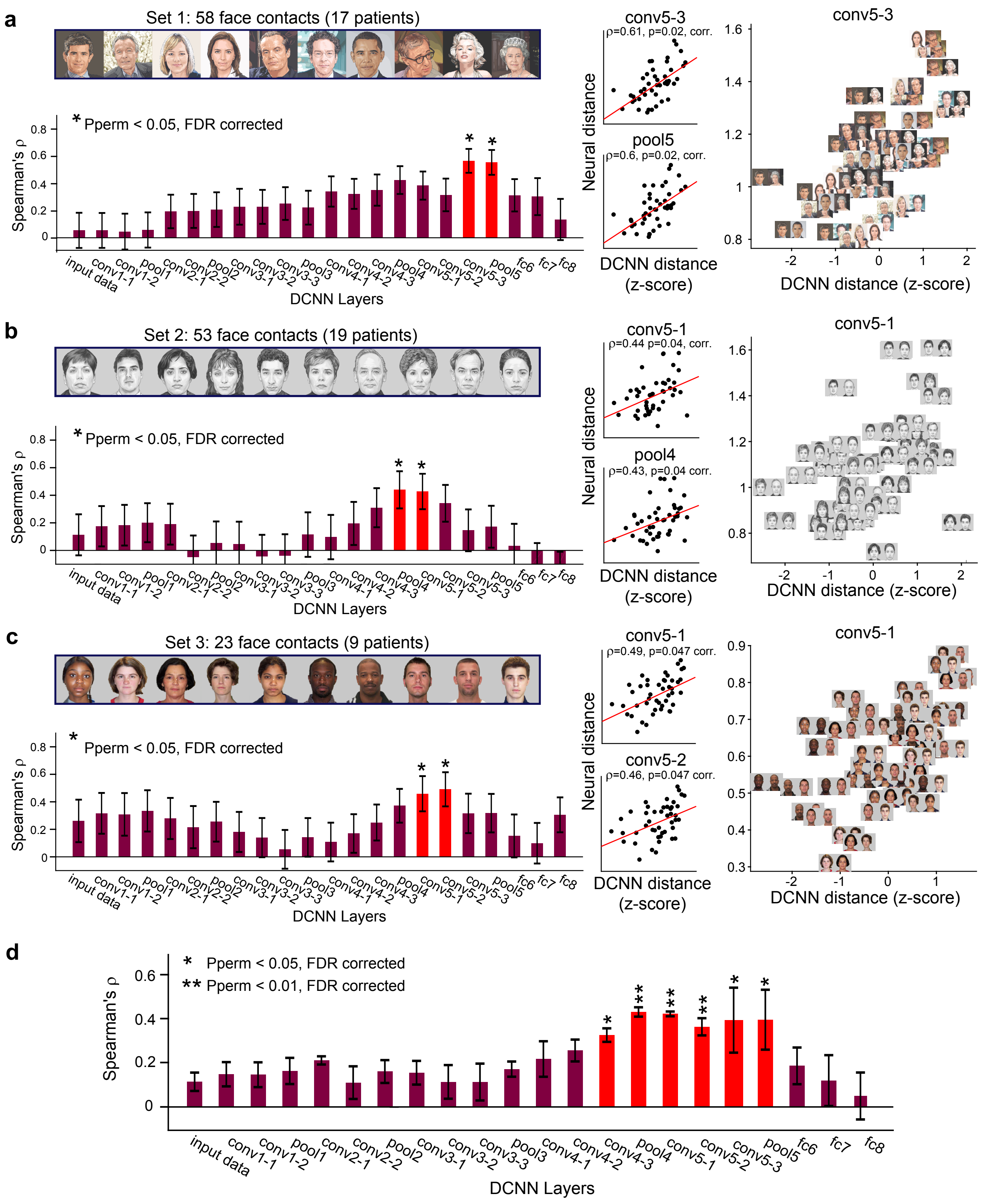

Supplementary Figure 7. Correlation between pair-wise distances in face-selective contacts and in DCNN layers for temporally averaged neural responses. The same analysis as presented in Fig. 2, with the difference of averaging the HFA response over both exemplar repetitions and time (50-500 ms), rather than averaging them only over exemplar repetitions. The observed correlations are highly similar to those received for the non-temporally averaged neural responses. Error bars denote image pairs bootstrap s.e.m. Faces used in set 3 (and shown here) were taken from the face data base by Minear, M. & Park, D.C.(2004). A lifespan database of adult facial stimuli. Behavior Research Methods, Instruments, & Computers. 36, 630-633.

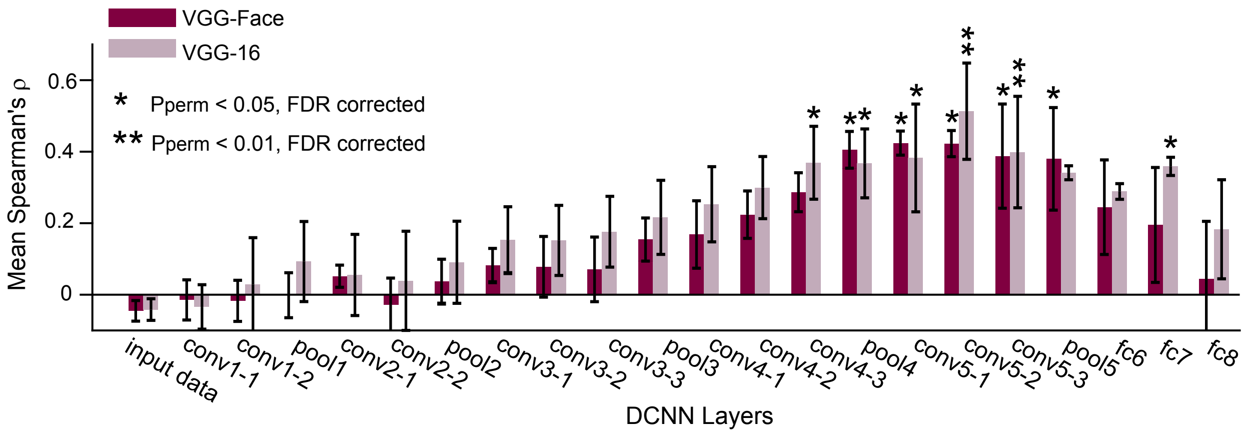

Supplementary Figure 8. Comparison of neural face-space match to VGG-Face and to VGG-16 DCNNs. The same analysis as presented in Fig. 2d, with the correlations to VGG-16 presented side by side with the correlations to VGG-Face. Error bars denote image pairs bootstrap s.e.m. P values were derived from permuting image labels 1,000 times and re-computing the weighted average correlation across the 3 sets (as in Fig. 2d), followed by a 5% FDR correction across layers, per network.

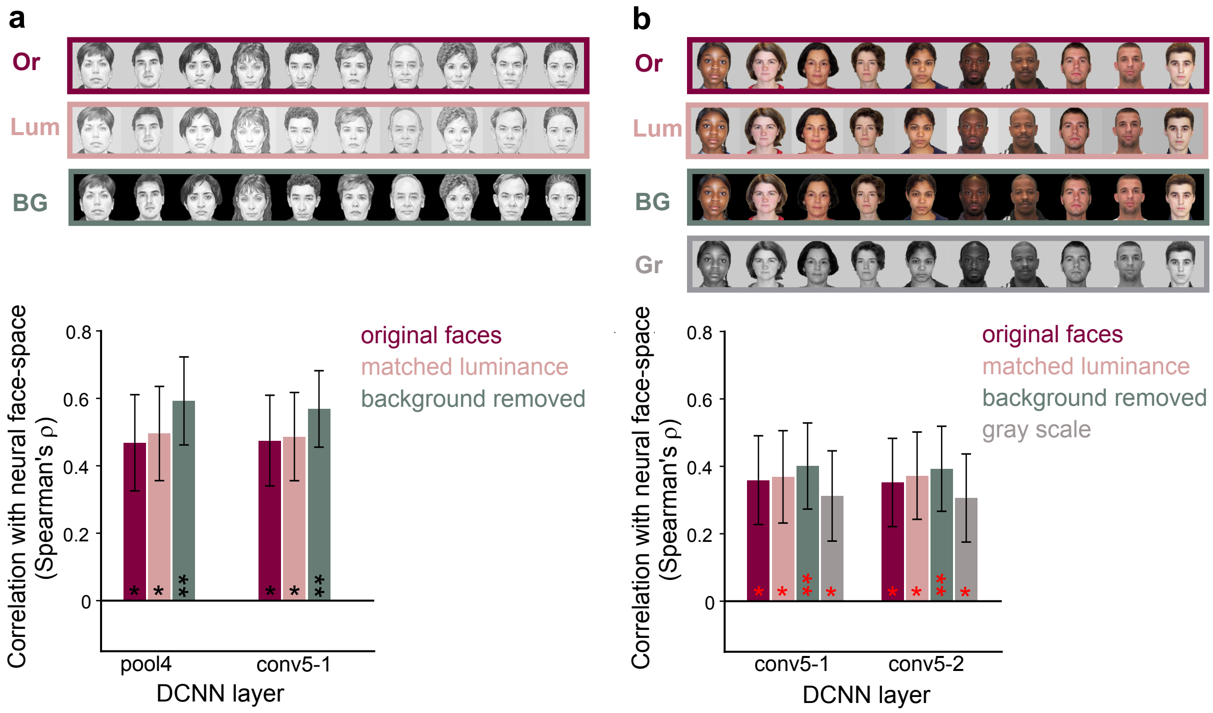

Supplementary Figure 9. The impact of low-level manipulations to face-exemplars presented to the DCNN on the match to neural face-space in sets 2 and 3. The same analysis as presented in Fig. 3 for set 1, applied here on set 2 (**a**) and set 3 (**b**). Since view-shifted and different appearance images of the exemplars in sets 2 and 3 were not available, we applied only the low-level manipulations. Note that gray-scale conversion was not applied on set 2 since images were originally gray scaled. *Pperm<0.05, **Pperm<0.01. Black asterisks denote significant correlations following 5% FDR correction across all network layers, whereas red asterisks denote significant correlation that did not survive FDR correction. Faces used in set 3 (and shown here in panel b) were taken from the face data base by Minear, M. & Park, D.C.(2004). A lifespan database of adult facial stimuli. Behavior Research Methods, Instruments, & Computers. 36, 630-633.

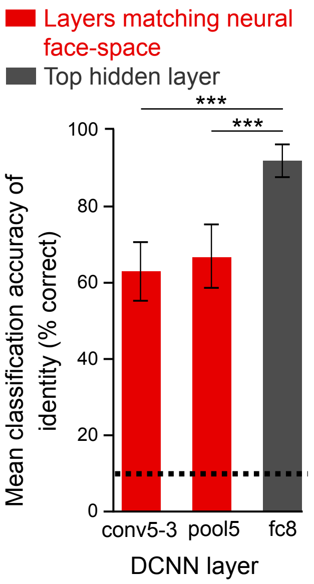

Supplementary Figure 10. Top hidden layer of the DCNN is robust to identity preserving face manipulations, whereas intermediate layers matching face-selective contacts are sensitive to such manipulations. Identity classification accuracy of a nearest neighbor classifier applied to the pooled images from all 4 conditions (original images and 3 high level manipulations presented in Fig. 3). Classification was performed 1,000 times, each time randomly assigning 10 test images of the 10 identities from the different conditions to a test set. Error bars are the s.e.m. of classification accuracy across the 10 identities. Dashed line denotes chance performance (10%). *** $p<0.001$, computed in a two samples t-test between performance distributions obtained from each layer.

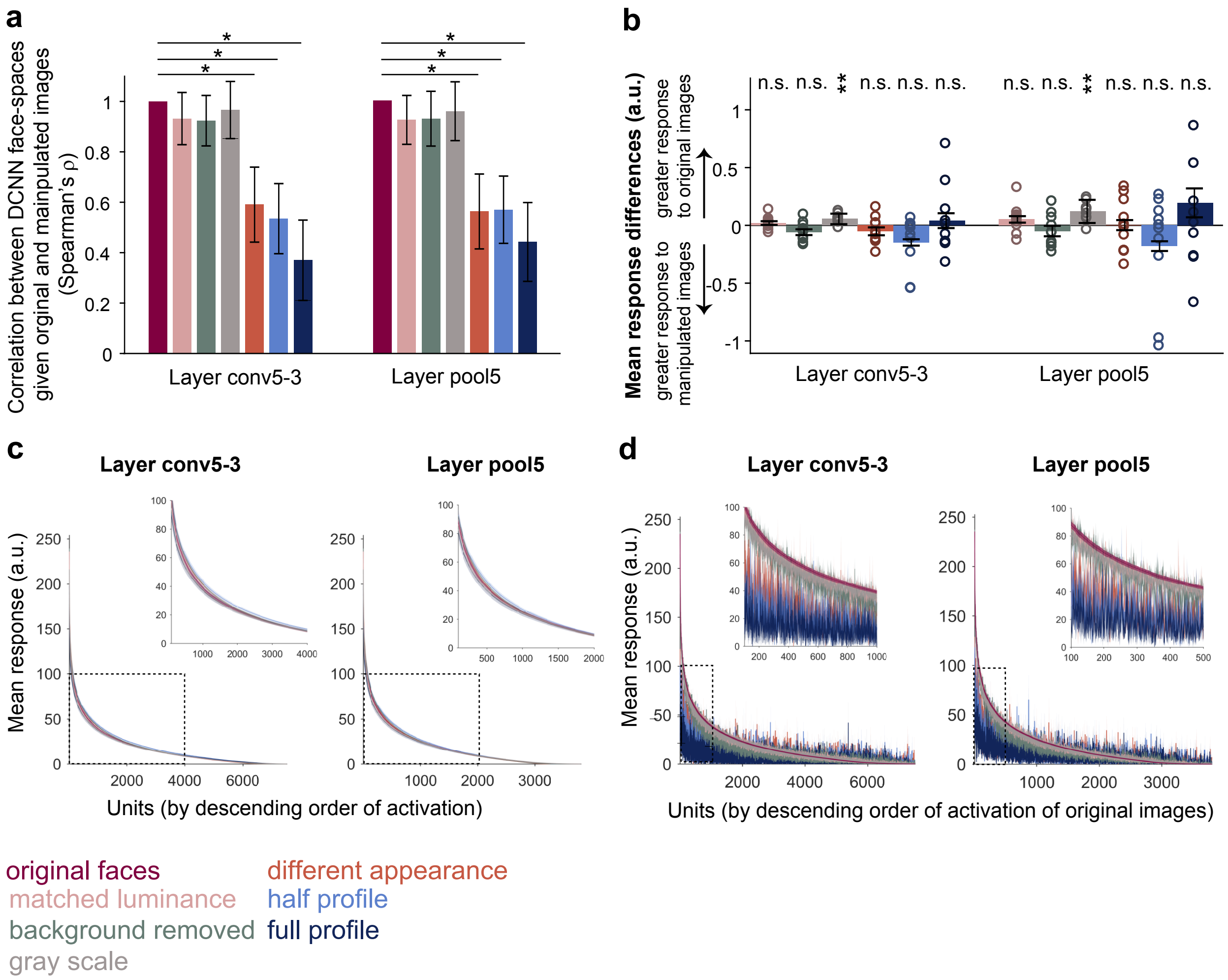

Supplementary Figure 11. The impact of image manipulations on pair-wise distances, amplitude and pattern of DCNN layers' activations. a The correlations between pair-wise distances given the original images as inputs and pair-wise distances given each of the manipulated sets presented in Fig. 3a as inputs was computed for the two VGG-Face layers that matched the neural face-space. Low level manipulations (matched luminance; background removal; and gray scale conversion) had minor impact on pair-wise distances, whereas high level identity-preserving manipulations markedly reduced the correlation. *p<0.05, image permutation test on correlation decrease. **b** The differences in mean amplitude of layer response following each of the six manipulations presented in Fig. 3a. Bar height is the difference between layer mean response to the original and manipulated image, averaged across the 10 exemplars from set 1. Circles denote single exemplars. Error bars denote ±1 s.e.m. ** p<0.01, uncorrected (Wilcoxon signed-rank tests). **c** Layers' activation profile to each of the 6 manipulated image sets and the original set. For each set, activations per image were sorted (left to right) by a descending order and averaged across images. Shaded areas denote ±1 s.e.m. Insets are enlarged fractions of the superimposed curves, delineated by the dashed rectangles. Note the similarity in the shape of the overall sorted amplitudes across conditions. **d** Similar to panel c, but here the order of activation vectors was kept fixed according to the sorted activation indices given the original images as input. The identity of responsive units follows a similar pattern to the that elicited by the original images for low-level manipulations, whereas patterns following high-level identity-preserving manipulations depart from the original pattern. Note that for panels b and c, x axis was truncated according to the number of responsive units given the original images set.

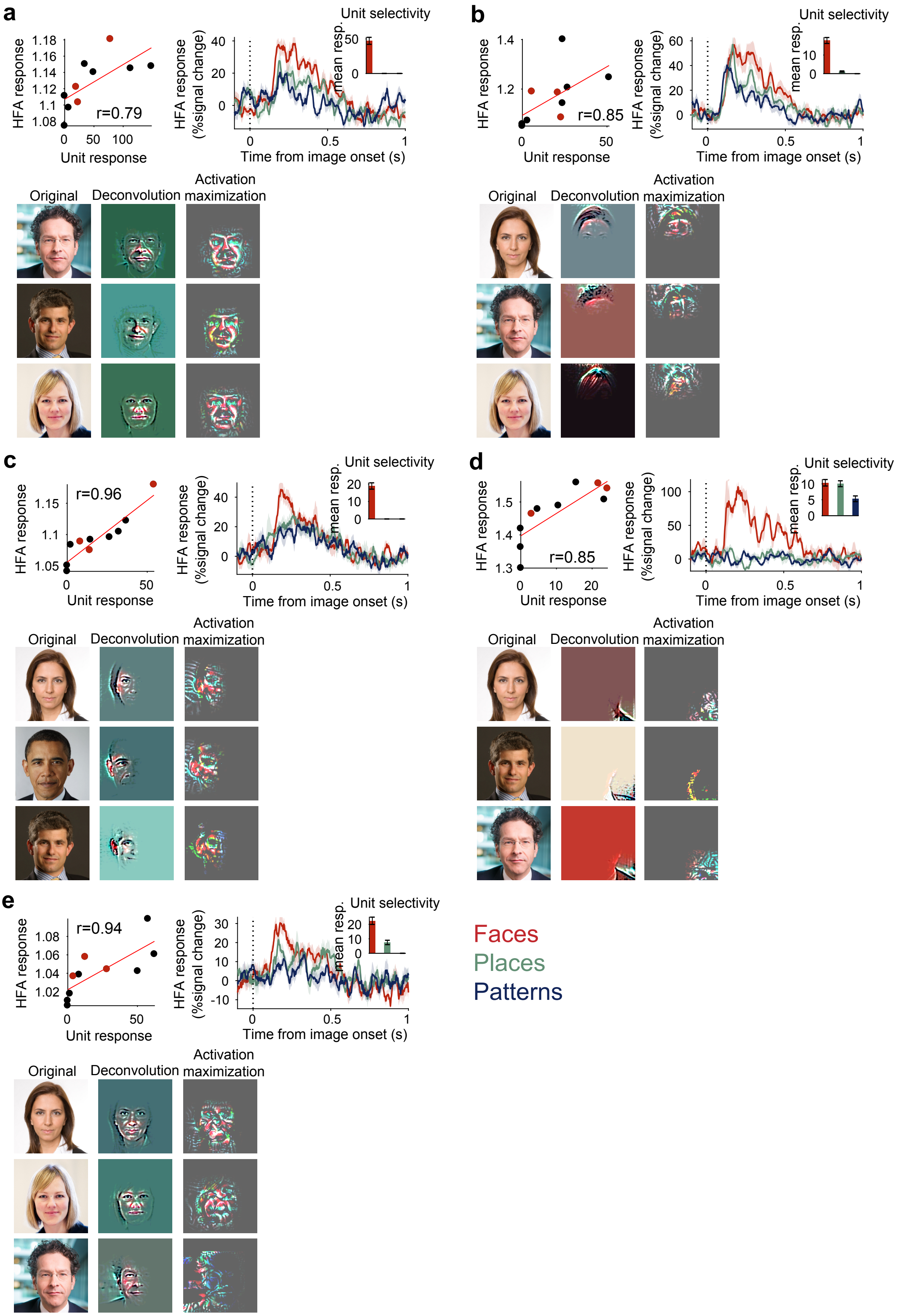

Supplementary Figure 12. Remaining model units detected with their receptive fields visualizations. The same analysis as presented in Fig. 4 for the remaining 5 model units detected, all in set 1. Copy rights holders: Christopher Falkenberg photo – Christopher Falkenberg; Barak Obama photo – Pete Souza, license link https://creativecommons.org/licenses/by/3.0/deed.en; Ida Auken photo – Johannes Jansson/norden.org, license link https://creativecommons.org/licenses/by/2.5/dk/deed.en; Gila Gamliel photo – https://en.wikipedia.org/wiki/Gila_Gamliel#/media/File:Gila_Gamliel.jpg, license link https://creativecommons.org/licenses/by-sa/3.0/deed.en.

Patient code	Sex	Age	Seizure Onset Zone(s)	Number of face contacts	Set 1	Set 2	Set 3
P051	F	26	LH: TP, Lateral Orbital Sulcus	5	-	V	-
P052	F	40	LH: Temporal pole, HPC	1	-	V	-
P056_imp2	F	30	RH: Lateral fissure, STG	2	-	V	-
P059	F	25	LH: Middle Temporal (STS), Parahippocampal Gyrus, Anterior Fusiform Gyrus, HPC, Insula	1	-	V	-
P061	M	22	RH: TP, Middle Temporal, HPC, IFG, MFG, SFG	3	-	V	-
P063	M	45	RH: Ventro Dorso Lateral, Pre-Central Sulcus	2	-	V	-
P070_imp2	M	23	RH: Insula, Middle Temporal (STS)	5	V	V	-
P071	M	23	LH: STG, HPC	2	-	V	-
P074	F	52	LH: AMYG, HPC	6	V	V	-
P075	M	24	LH: TP, Parahippocampal Gyrus	1	V	V	-
P078_imp2	M	25	LH: AMYG	6	V	V	-
P078_imp3	M	25	LH: AMYG	1	V	V	-
P079	M	40	LH: AMYG, HPC, TP	1	-	V	-
P082	M	45	LH: HPC , Parahippocampal Gyrus	3	V	V	-
P083	F	34	LH: TP, Inferior Temporal	6	V	-	-
P089	M	21	RH: Medial Temporal, MOG, Parieto-Occipital Sulcus, MTG<br>LH: HPC	2	-	V	-
P092	M	28	LH: HPC , Lateral Temporal	7	V	V	-
P094	M	29	LH: AMYG	2	V	V	-
P096	M	50	RH: Parieto-Occipital-Sulcus	6	V	-	-
P097	M	38	LH: AMYG, HPC, Parahippocampal Gyrus, Insula	2	V	V	-
P098	F	44	LH: HPC, Anterior Cingulate Gyrus, AMYG	1	V	V	-
P101	M	49	LH: AMYG, HPC	5	V	-	-
P103	F	29	RH: ITG, Fusiform Gyrus	2	-	V	-
P108	M	46	LH: Gyrus Rectus, Putamen	2	-	V	-
P110	F	29	LH: Anterior Parahippocampal Gyrus	1	V	-	-
P112	M	59	LH: AMYG, HPC	3	-	-	V
P116	M	41	LH: HPC<br>RH: AMYG	1	-	-	V
P117	F	56	LH: Middle Temporal, inferior Parietal Gyrus	1	-	-	V
P120	F	37	Inferior Fusiform Gyrus	6	-	-	V
P120_imp2	F	37	RH: MTG	4	-	-	V
P121	M	20	RH: AMYG, HPC, Cuneus, Precuneus	3	V	-	V
P126	M	43	LH: AMYG, HPC, Parahippocampal Gyrus, Collateral Sulcus	2	-	-	V
P129	M	17	LH: Lateral Fissure, STS, STG	1	V	-	V

Supplementary Table 1. Demographic, clinical and experimental details of patients. LH /RH – left/right hemisphere; IFG/MFG/SFG – Inferior/Middle/Superior Frontal Gyrus; ITG/MTG – Inferior/Middle Temporal Gyrus; MOG/SOG – Middle/Superior Occipital Gyrus; STS/STG – Superior Temporal Sulcus/Gyrus; TP – Temporal Pole; AMYG – Amygdala; HPC – Hippocampus. Patients were coded according to the order of admission to surgery. Since not all patients participated in the experiment or were found to have face selective contacts, coding is not consecutive. imp2/imp3 suffix in patient code denotes cases of several implants in the same patient.
